# Frequency of anticancer drug use at the end of life: a scoping review

**DOI:** 10.1007/s12094-023-03234-1

**Published:** 2023-06-08

**Authors:** Endre Szigethy, Rosario Dorantes, Miguel Sugrañes, Meisser Madera, Ivan Sola, Gerard Urrútia, Xavier Bonfill

**Affiliations:** 1 PhD Programme in Biomedical Research Methodology and Public Health, Universitat Autònoma de Barcelona, Barcelona, Spain; 2 Centre Assistencial Dr. Emili Mira, Parc de Salut Mar, Santa Coloma de Gramenet, Barcelona, Spain; 3 School of Medicine, Universitat Autònoma de Barcelona, Barcelona, Spain; 4 Research Department, Faculty of Dentistry, University of Cartagena, Cartagena, Colombia; 5 Iberoamerican Cochrane Centre, Biomedical Research Institute Sant Pau (IIB Sant Pau), CIBER Epidemiología y Salud Pública (CIBERESP), Universitat Autònoma de Barcelona, Barcelona, Spain; 6 Epidy Kft, Debrecen, Hungary

**Keywords:** Neoplasms, Anticancer agents, Terminal care, Medical overuse

## Abstract

**Purpose:**

Anticancer drug use at the end of life places potential extra burdens on patients and the healthcare system. Previous articles show variability in methods and outcomes; thus, their results are not directly comparable. This scoping review describes the methods and extent of anticancer drug use at end of life.

**Methods:**

Systematic searches in Medline and Embase were conducted to identify articles reporting anticancer drug use at the end of life.

**Results:**

We selected 341 eligible publications, identifying key study features including timing of research, disease status, treatment schedule, treatment type, and treatment characteristics. Among the subset of 69 articles of all cancer types published within the last 5 years, we examined the frequency of anticancer drug use across various end of life periods.

**Conclusion:**

This comprehensive description of publications on anticancer drug use at end of life underscores the importance of methodological factors when designing studies and comparing outcomes.

**Supplementary Information:**

The online version contains supplementary material available at 10.1007/s12094-023-03234-1.

## Introduction

Palliative anticancer drug use is reported to prolong survival and relieve disease-related symptoms for some patients with incurable cancer [1, 2]. Conversely, other studies have reported a marginal impact of anticancer therapy on life expectancy and showed an unclear relationship between the use of this treatment and quality of life [3, 4]. Furthermore, the potential benefits of using anticancer drugs in terminally ill cancer patients are disputed because cancer therapy might threaten patients’ quality of life and even survival, and also imposes a significant cost on the healthcare system. Moreover, an increasing number of articles claim that near the end of life, anticancer treatment is associated with poorer quality of life [5–7] and does not improve survival [8, 9]. In this review, the term “end of life” is used referring to final days, weeks, months in a person’s life in which it is medically obvious that death is imminent or a terminal moribund state cannot be prevented [10].

Anticancer drug use towards the end of life is highly prevalent worldwide and increasing in some countries [11]. A single centre study in Korea reported that, the proportion of patients receiving chemotherapy in the last month of life was 25.7% in 2000, 32.7% in 2005, and continued to increase to 44.2% in 2010 [12]. In the USA, between 1993 and 1996, the proportion of patients receiving chemotherapy within the 2 weeks before death rose from 13.8% to 18.5%, while recent studies reported stagnation in the USA [13–15]. The trend and magnitude of end of life anticancer treatment frequency may be explained in part by the rapidly evolving treatment options in oncology, like immunotherapy and newer targeted treatments, which have broadened the therapeutic landscape. Recent reviews on resource utilization in end of life cancer patients show great diversity in settings, methods, and outcomes across publications [11, 16, 17].

These differences are more evident when defining the study population. While in retrospective population-based studies, which include all cancer patients in the analysis, regardless of previous treatments received or stage of cancer, they typically reported a lower frequency of end of life cancer treatment [15, 18, 19]. Conversely, research studies that enrolled patients with advanced cancer or those who had undergone cancer treatment in the past reported substantially higher frequencies of end of life cancer treatment in the same period [20, 21].

The goal of this article is to explore the methods and outcomes used by the studies, and along these differences, describe the current magnitude of anticancer treatment at end of life. These objectives lend themselves to a scoping review approach because they aim to identify knowledge gaps, clarify concepts, and scope the literature related to cancer treatment at end of life. Scoping reviews are particularly useful for mapping the existing literature in a specific field, allowing researchers to compare the methods and outcomes used by various studies [22]. In this case, a scoping review can provide a comprehensive overview of the current magnitude of anticancer treatment at end of life, highlighting differences in methods and outcomes across studies. Furthermore, this scoping review can inform decision-making and set research agendas to identify areas that require further investigation and inform future systematic reviews or primary research on this topic [23].

## Methods

### Study identification

Scoping review was conducted based on the methodology proposed by JBI PRIMSA ScR [24–26], by undertaking a systematic search in Medline (via PubMed) and Embase (via Embase) literature databases using combinations of keywords (namely: ‘aggressive’, ‘end of life’, ‘last days/weeks/months’, ‘until/near death’, ‘late/end stage’, ‘days/weeks/months of life’, ‘antineoplastic’, ‘anticancer’, ‘chemotherapy’, ‘hormone/immune/biological’, ‘cancer/malignancy/neoplasm/tumour and care/drug/treatment/therapy’). The search strategy developed for Medline is displayed in online Appendix 1. The research protocol is available upon request.

### Eligibility criteria

Publications reporting observational primary research studies on end of life anticancer drug use with a sample size of over 100 patients were included. Using this arbitrary sample size criterion, the probability of random error of individual estimates arising from low sample sizes is reduced. Due to the specific disease characteristics and treatment protocols of children, this review excluded studies reporting results only in paediatric patients. Moreover, randomized controlled trials were not included in this review, as the explanatory designs might lead to an increased rate of anticancer drug administration at end of life.

To explore the methods and outcomes used by the studies on end of life anticancer drug use, all articles published prior to November 30, 2020 were included in this review, which was the date of the most recent search. To provide up-to-date information on frequencies of end of life anticancer drug use in patients with any cancer types (non-specific for tumour type or location), articles published in the last 5 years (i.e. after January 01, 2016) were included.

### Selection of studies

Two authors (ES and RD) screened the search results (titles, abstracts) against the eligibility criteria. Potentially relevant full-text articles were selected. Discrepancies across screeners were resolved through discussion or by a third reviewer (MM). The author team selected articles for review based on their linguistic proficiency in English, Spanish, French, German, Hungarian, Italian, Russian, and Chinese.

### Data extraction

As per the double data extraction method, two reviewers independently obtained data from each article and resolved discrepancies by consensus. In total, 17 undergraduate medical students were trained to collaborate in the second data extraction process and checked the accuracy of coded variables on all data-entry forms. The data extraction process involved the retrieval of several key variables from the included publications. These variables included the study period, study design, data source, country, patient characteristics (number of patients, age distribution, and gender distribution), cancer stage, treatment status, cancer type(s), inclusion and exclusion criteria, treatment type (as defined by the authors), all end of life periods reported, and the frequency of patients receiving anticancer treatment during the specified periods. By systematically extracting and analysing these variables, we aimed to gain a comprehensive understanding of the patterns and frequencies of anticancer treatment utilization at the end of life across different studies.

### Analysis and reporting

This scoping review adheres to the reporting standards outlined by the Preferred Reporting Items for Systematic Reviews and Meta-Analyses extension for Scoping Reviews (PRISMA-ScR) criteria [27]. The authors have followed the PRISMA-ScR checklist to ensure the completeness and transparency of their review methodology and reporting. The publications were clustered according to their methodological characteristics in respect of study design (prospective or retrospective), data source (hospital-based medical records or population based data, including registries, claims data, and administrative datasets), disease status of enrolled patients (all cancer stages or advanced cancer patients), patient population definition (the denominator of the proportion includes either all cancer patients or previously treated cancer patients), treatment initiation (start of new anticancer regimen or receiving any anticancer treatment), and anticancer treatment type (as defined by the individual articles, including biologics, chemotherapy, hormonal/endocrine therapy, immunotherapy, targeted therapy, and systemic anti-cancer treatment). As in any scoping review, no attempts have been made for performing data analysis. Instead, this article describes the key study features and results across the included studies, analysed data items by quantifying text and doing frequency counts of data extraction items with narrative and tabular presentation of results.

## Results

### Studies identified for review

**Fig. 1:**
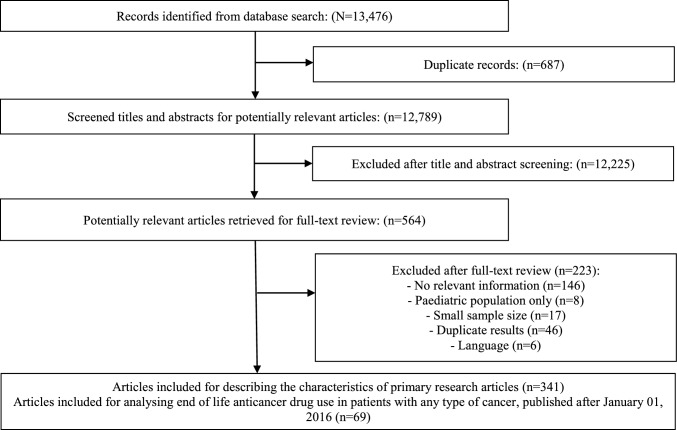
Scoping review flowchart

A comprehensive search was completed in October 2018 and updated in November 2020 resulting in a total of 13,476 articles. After removing duplicates, 12,789 publications were screened for eligibility by titles and abstracts. Subsequently, 341 publications were finally included to explore and describe the various methods and outcomes utilized by the studies, in accordance with the objectives of this scoping review. Furthermore, a subset of these articles (n = 69), those published in 2016 or later, were selected to analyse the current magnitude of end of life anticancer drug use in patients with any type of cancer (Fig. 1). Articles meeting the eligibility criteria that did not satisfy the aforementioned subset requirements, including those centred on specific cancer types, were preserved for potential inclusion in future publications. The reference list of included 341 publications and its subset of 69 articles is displayed in online Appendix 2.

### Characteristics of primary research articles reporting end of life anticancer drug use

**Fig. 2:**
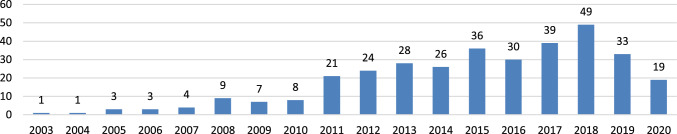
Number of articles by publication date (any type and specific types of cancer N = 341)

Analysis was performed on the characteristics of primary research articles reporting end of life anticancer drug use across 341 eligible studies to explore the common methods and outcomes employed by these studies. The number of publications increased over time, ranging from one publication in 2003 and peaking at 49 articles in 2018, although this upward trend did not continue in the in the subsequent years (Fig. 2). Given that the article search for this review concluded in November 2020, it is important to acknowledge that the inclusion of articles published in the same year is incomplete. Most of the studies (43.4%, n = 148) were from North America, with the remaining studies from Europe (34.3%, n = 117), Asia (16.1%, n = 55), Australasia (4.4%, n = 15), South America (1.5%, n = 5), Africa (0.3%, n = 1). About 40% of selected studies were abstract (Table 1).

**Table 1:** Characteristics of articles describing end of life anticancer drug use (any type and specific types of cancer N = 341)

	Proportion of articles (number of articles)
**Region**	
Africa	0.3% (n = 1)
Asia	16.1% (n = 55)
Australasia	4.4% (n = 15)
Europe	34.3% (n = 117)
North America	43.4% (n = 148)
South America	1.5% (n = 5)
**Article type**	
Abstract	40.3% (n = 151)
Full-text article	59.7% (n = 224)

N = total number of articles

### Methodological features of selected publications

Most studies (80.9%; n = 276) retrospectively assessed anticancer drug use at the end of life in deceased cancer patients, while 19.1% (n = 65) were prospective studies, enrolling alive patients (Table 2). Among the whole 341 studies, 89.4% (n = 305) reported on any previously initiated anticancer treatment, 0.6% (n = 2) reported on starting a new anticancer regimen at end of life, and 10.6% (n = 36) reported both any previous treatment and the start of a new anticancer regimen. Chemotherapy was the most frequently reported anticancer medication (90.6%; n = 309), followed by targeted therapies (6.2%; n = 21), hormonal/endocrine therapies (2.6%; n = 9), immunotherapies (2.3%; n = 8), and biologics (1.2%; n = 4). In addition to these specific anticancer treatment types, the use of any systemic anti-cancer treatment (SACT) was assessed in 7.0% (n = 24) of publications. Individualizing them was impossible due to the variations in SACT definitions among studies. Some publications evaluated all kinds of anticancer drugs as SACT, others focused solely on chemotherapies and targeted therapies (Table 2).

**Table 2:** Methodological features of included publications (any type and specific types of cancer N = 341)

	Articles describing end of life anticancer treatment patterns
**Study design**	
Retrospective (deceased patients)	80.9% (n = 276)
Prospective (all cancer patients)	19.1% (n = 65)
**Treatment during end of life period**^a^	
Any previously initiated anticancer treatment	89.4% (n = 305)
Starting new anticancer regimen	0.6% (n = 2)
Any previously initiated anticancer treatment and starting new anticancer regimen	10.6% (n = 36)
**Treatment type**^a^	
Systemic anti-cancer treatment (all)	7.0% (n = 24)
Biologics	1.2% (n = 4)
Chemotherapy	90.6% (n = 309)
Hormonal/endocrine therapy	2.6% (n = 9)
Immunotherapy	2.3% (n = 8)
Targeted therapy	6.2% (n = 21)

n = number of articles; N = total number of articles

^a^Overlap between categories is possible

**Fig. 3:**
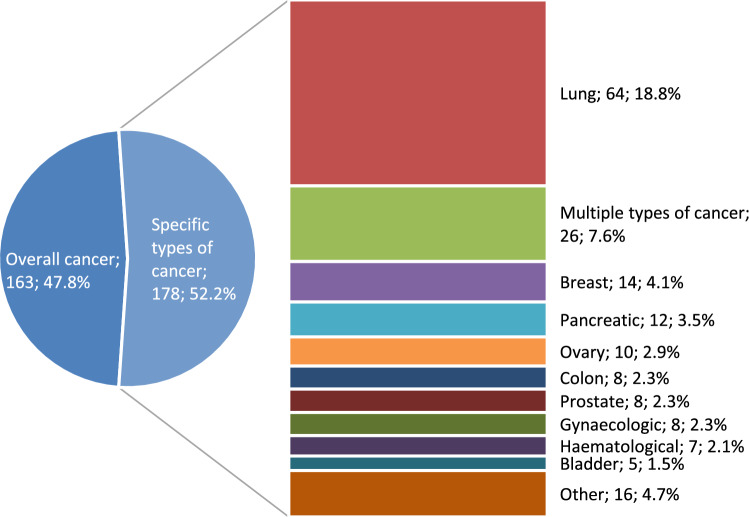
The cancer-type specific distribution of articles describing end of life anticancer treatment patterns (any type and specific types of cancer N = 341)

Around half of the studies (47.8%, n = 163) described the frequency of anticancer drug use at the end of life in patients with any type of cancer aggregating all types of malignancies. More than one specific type of cancer (multiple) was reported (up to 9 types of cancer) in 7.6% (n = 26), whereas a single cancer type was assessed in 44.6% (n = 152). The most common cancer type was lung cancer (n = 64), followed by breast (n = 14), pancreatic (n = 12), and ovarian cancer (n = 10). Anticancer drug use at the end of life was assessed for 16 further cancer types, with fewer than 10 publications for each type (Fig. 3).

### Frequency of anticancer therapies at the end of life

In the subset of articles (N = 69) analysed to explore the extent of end of life treatment reported across all cancer types within the last 5-year period, 27.5% (n = 19) were population-based studies, all of which were retrospective. Among the hospital-based studies (72.5%, n = 50), 14.0% (n = 7) were prospective, while 86.0% (n = 43) applied a retrospective design. With the exception of one population-based study, all others enrolled over 2,000 patients, with sample sizes ranging from 835 to 516,244 patients. Among the hospital-based studies, the majority (54%, n = 27) included less than 500 patients. The majority of population-based studies (89.5%, n = 17) analysed the end of life treatment of all cancer patients, while in hospital-based studies, 66.0% (n = 33) included all cancer patients, and 34% focused specifically on patients with advanced-stage cancer. Around a quarter of the population-based studies (26.3%, n = 5) focused on previously treated patients when calculating the occurrence of end of life anticancer treatment. In contrast, more than half of the hospital-based studies (52.0%, n = 26) enrolled patients who had received treatment in the past (Table 3).

**Table 3:** Methodological features of publications describing end of life treatment (patients with any type of cancer, published after January 01, 2016; N = 69)

	Population-based studies (N = 19)	Hospital-based studies (n = 50)
**Study design**		
Prospective	0% (n = 0)	14.0% (n = 7)
Retrospective	100% (n = 19)	86.0% (n = 43)
**Study sample (patients)**		
> 2000	94.7% (n = 18)	8.0% (n = 4)
500–2000	5.3% (n = 1)	38.0% (n = 19)
100–500	0% (n = 0)	54.0% (n = 27)
**Disease status**		
All cancer stages	89.5% (n = 17)	66.0% (n = 33)
Advanced stages	10.5% (n = 2)	34.0% (n = 17)
**Treatment characteristics**		
Treated and non-treated patients	73.7% (n = 14)	48.0% (n = 24)
Treated patients	26.3% (n = 5)	52.0% (n = 26)

n = number of cases; N = total number of articles

End of life periods analysed across the studies ranged from 3 days to 6 months. The most commonly applied end of life periods were the last 2 weeks of life (66.7%, n = 46), the last month of life (63.8%, n = 44), and the last 3 months of life (21.7%, n = 15). Multiple periods were reported in 42.0% (n = 29) of the studies, and 11.6% (n = 8) applied 4 or more different periods to determine the prevalence of end of life anticancer drug use. In publications reporting one end of life period, the last 2 weeks of life was used most often (52.5%, n = 21). Starting a new anticancer regimen in the final months of life was assessed by 13.1% (n = 9) of studies, and all of them reported at least one further end of life period.

**Fig. 4:**
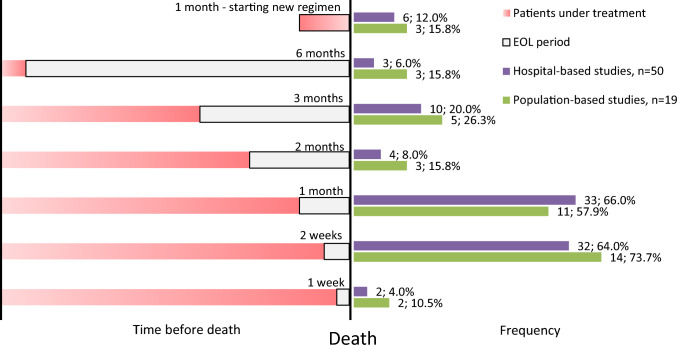
End of life treatment periods analysed and their frequencies in the publications; only categories with more than one publication are presented (patients with any type of cancer, published after January 01, 2016; N = 69)

In the hospital-based studies (n = 50), the frequencies of reporting the last month of life and the last 2 weeks of life were similarly high, with proportions of 66.0 and 64.0%, respectively. However, in population-based studies, the last 2 weeks of life was more frequently reported (73.7%) compared to the last month of life (57.9%). Additionally, the other end of life periods were investigated more frequently in population-based studies compared to hospital-based studies, as shown in Fig. 4.

In the 46 studies assessing end of life treatment in the last 2 weeks of life, the frequency of anticancer drug use ranged between 0.2 and 29%. In the final month of life, the reported range of frequencies across publications was 1 to 56%. The 3-month end of life period was used in 15 articles, showing even greater variability in treatment rates, the lowest estimates reported 3% while the highest was 98%. The final 2 months of life were described in 7 studies, with frequency ranging between 2 and 30%, while the last 6 months were reported in 6 studies (range 16–79%). The sixth most commonly described period was the final week of life (n = 3), when between 4 and 7% of patients received anticancer treatment. It was uncommon to report end of life anticancer drug use in the last 6 days or less, in the last 3 weeks, or in the last 4 or 5 months. Frequencies of end of life treatments with at least two published results are shown in Table 4. The frequency of initiating a new anticancer regimen during the last month before death was examined in a total of 7 studies, with reported rates ranging from 2 to 21%. One study reported a 10% frequency of initiating a new regimen within the last 2 weeks before death, while another study reported a frequency of 9% within the last 2 months before death.

**Table 4:** Reported frequency of anticancer drug use at the end of life (patients with any type of cancer, published after January 01, 2016; N = 69)

End of life period	1 week	2 weeks	1 month	2 months	3 months	6 months
Total	4–7% (n = 4)	0–29% (n = 46)	1–56% (n = 44)	2–30% (n = 7)	3–98% (n = 15)	16–79% (n = 6)
**Publication**						
Abstract (n = 21)	7% (n = 1)	1–26% (n = 14)	5–56% (n = 15)	28% (n = 1)	19–98% (n = 5)	64% (n = 1)
Full text (n = 48)	4–7% (n = 3)	0–29% (n = 32)	1–45% (n = 29)	2–30% (n = 6)	3–75% (n = 10)	16–79% (n = 5)
**Data source and design**						
Hospital-based (n = 50), including:	7% (n = 2)	0–29% (n = 32)	2–56% (n = 33)	3–30% (n = 4)	5–98% (n = 10)	52–79% (n = 3)
Prospective (n = 7)	–	1–5% (n = 4)	5–28% (n = 6)	3–30% (n = 3)	5–36% (n = 3)	–
Retrospective (n = 43)	7% (n = 2)	0–29% (n = 28)	2–56% (n = 27)	25% (n = 1)	19–98% (n = 7)	52–79% (n = 3)
Population-based (n = 19)	4–7% (n = 2)	1–24% (n = 14)	1–31% (n = 11)	2–28% (n = 3)	3–39% (n = 5)	16–46% (n = 3)
**Study sample (patients)**						
> 2000 (n = 22)	4–7% (n = 2)	1–24% (n = 17)	1–31% (n = 14)	2–28% (n = 4)	3–53% (n = 6)	16–79% (n = 4)
500–2000 (n = 20)	7% (n = 1)	0–29% (n = 11)	2–45% (n = 12)	3–30% (n = 2)	5–67% (n = 4)	–
100–500 (n = 27)	7% (n = 1)	1–27% (n = 18)	5–56% (n = 18)	11% (n = 1)	13–98% (n = 5)	52–64% (n = 2)
**Disease status**						
All cancer stages (n = 50)	4–7% (n = 2)	0–29% (n = 34)	1–56% (n = 32)	2–28% (n = 5)	3–98% (n = 11)	16–79% (n = 4)
Advanced stages (n = 19)	7% (n = 2)	1–24% (n = 12)	2–42% (n = 12)	3–30% (n = 2)	5–58% (n = 4)	16–52% (n = 2)
**Treatment characteristics**						
Treated and non-treated patients (n = 38)	4–7% (n = 3)	0–29% (n = 26)	1–56% (n = 22)	2–28% (n = 3)	3–98% (n = 8)	46% (n = 1)
Treated patients (n = 31)	7% (n = 1)	1–22% (n = 20)	5–42% (n = 22)	3–30% (n = 4)	13–75% (n = 7)	16–79% (n = 5)
**Treatment type**						
SACT (n = 18)	7% (n = 1)	2–27% (n = 8)	5–35% (n = 13)	18% (n = 1)	18–58% (n = 4)	–
Biologics (n = 1)	–	1% (n = 1)	1% (n = 1)	2% (n = 1)	3% (n = 1)	–
Chemotherapy (n = 54)	4–7% (n = 3)	0–29% (n = 40)	5–56% (n = 32)	10–30% (n = 6)	13–98% (n = 12)	16–79% (n = 6)
Hormonal/endocrine therapy (n = 2)	–	1% (n = 1)	5% (n = 1)	3% (n = 1)	5% (n = 1)	–
Immunotherapy (n = 3)	–	2–3% (n = 2)	7–28% (n = 2)	–	20% (n = 1)	–
Targeted therapy (n = 4)	–	1–5% (n = 4)	2–5% (n = 3)	–	19% (n = 1)	–
**Region**						
Africa (n = 1)	–	–	45% (n = 1)	–	–	–
Asia (n = 11)	7% (n = 1)	2–24% (n = 9)	5–42% (n = 7)	25% (n = 1)	75% (n = 1)	16% (n = 1)
Austral-Pacific (n = 6)	–	2–4% (n = 2)	5–31% (n = 6)	–	–	–
Europe (n = 27)	4–7% (n = 3)	2–27% (n = 18)	1–45% (n = 20)	2–30% (n = 5)	3–67% (n = 12)	46–79% (n = 2)
North America (n = 20)	–	1–26% (n = 14)	7–56% (n = 8)	13% (n = 1)	18–98% (n = 2)	18–64% (n = 3)
South America (n = 4)	–	0–29% (n = 3)	2–13% (n = 2)	–	–	–

n=Number of articles; N = total number of articles; SACT = systemic anti−cancer treatment

Stratified results of different end of life treatment periods showed little variation across study settings. Population-based studies have lower range of end of life drug use frequencies compared to hospital-based studies, for all periods. Prospective studies within the hospital-based category tend to have narrower ranges for most end of life periods compared to retrospective studies, suggesting that prospective designs may is associated with more focused and standardized methodology approaches. Publications with larger study sample sizes report lower frequencies of end of life drug use. This trend is particularly consistent in studies with sample sizes over 2000 patients, where lower frequencies are observed across all end of life periods compared to studies with sample sizes below 500 patients. Studies which investigated end of life drug use in advanced cancer patients found lower frequencies in almost every periods compared to studies which enrolled all cancer stages patients. End of life cancer treatment patterns vary depending on the disease stage, with lower frequencies observed in advanced cancer patients across almost every end of life period. Studies that focus solely on previously treated patients have reported lower rates of drug use, while studies that specify previously treated and non-treated patients as the denominator report higher rates in most end of life periods. Targeted therapies, immunotherapies, hormonal therapies, and biological treatments were used in a lower proportion of patients across all end of life periods, compared to chemotherapies or SACTs (Table 4). The analysis of end of life treatment periods across different regions revealed notable geographical differences in end of life anticancer treatment frequencies. Europe accounted for the largest number of estimates regarding end of life anticancer drug use, encompassing a range of frequencies within different periods that align with those observed in other regions. Following closely, North America emerged as the second most represented region, demonstrating comparable frequencies across various end of life periods. Similarly, Asia exhibited a notable volume of publications, presenting frequencies of end of life anticancer drug use that were consistent with those observed in other regions. Within the Austral-Pacific region, data were available for the last 2 weeks and last month of life, showing lower frequencies in comparison to the mentioned regions. Likewise, South America reported a lower proportion of patients receiving anticancer drug in the final month of life, while the frequencies in the last 2 weeks were comparable to those observed in other regions. A single publication from Africa reported a high frequency of end of life anticancer drug use in the final week of life (Table 4).

## Discussion

### Main findings of the study

Recent decades have seen an increasing scientific interest in the use of anticancer therapies at end of life. This trend is presumably fuelled by the continuously growing scientific knowledge on diverse cancer types and the expanding range of therapeutic options available, which has a large impact on the overall treatment journey [28–32]. The benefits of end of life cancer therapies are debated, with contradictory findings on their effect on survival and quality of life [1, 3, 4, 8]. To address concerns regarding cancer therapies at the end of life, it is imperative to accurately quantify the extent of the phenomenon. However, this task presents considerable challenges due to the inherent heterogeneity in scientific approaches employed and the subsequent variability in the obtained results. The multidimensional complexity of this issue includes objective and subjective factors that interact over time, contributing to the elusive nature of the end of life concept [33, 34]. Thus, analysing the methodological characteristics of relevant research studies is a prerequisite to describing and understanding the current trends of cancer therapies at the end of life.

This scoping review on primary research studies reporting end of life anticancer drug use had a dual purpose. Firstly, it aimed to explore and describe the characteristics of these primary research articles, leading to the identification of key features in 341 eligible publications, including the timing of research, disease status, definition of treatment schedule, treatment type, and treatment characteristics. Secondly, to achieve a comprehensive understanding of the frequency of end of life anticancer drug use, we restricted our analysis to 69 publications concerning patients with any type of cancer published within the last 5 years. This approach facilitated detailed analyses and yielded insights into the current extent of end of life anticancer drug use.

The broader set of 341 articles enabled a comprehensive depiction of the methodologies and outcomes employed in these primary research studies. The number of publications showed that this topic has attracted more attention in recent decades, as from 2015 there was a further increase in the number of articles, with a peak in 2018. The geographical distribution of identified articles indicates that anticancer drug use at end of life is mostly reported from developed countries [35]. Probably in relation to the country’s economic status, cancer patients in developing regions are less exposed to potential overtreatment and more frequently face undertreatment due to difficulties in accessing well proven standard therapies, coupled with a lower capacity for conducting health research. From the other hand, resource constraints may also limit the quantity of research conducted in low and middle income countries, moreover, it is possible that there are other factors contributing to the disparities in the number of publications. These factors could include differences in research priorities, healthcare infrastructure, access to specialized cancer centres, and cultural attitudes towards end of life care [35].

One out of seven was a prospective study. Although this design may provide further detailed insights into the evolution of end of life anticancer drug use, in our review, the prospective studies had smaller sample sizes than the retrospective studies, which increases the likelihood of random error. Conversely, retrospective studies, which accounted for the vast majority of the studies identified, can be more affected by misclassification bias. The identified literature is dominated by hospital-based studies, which had smaller sample sizes compared to the other studies and which included every prospective study.

Furthermore, prospective and retrospective studies have methodological differences related to the timing of the research. This impacts how the denominator of the frequency is determined and, as a result, makes direct comparisons between the two study types challenging. In prospective studies, the denominator of the frequency ratio includes the entire patient population, including those still alive at the time of measurement. On the other hand, retrospective studies employ a different methodology where treatment frequency is measured among patients who have died. In this case, the denominator is restricted to the deceased patient population. Understanding and recognizing these methodological disparities is crucial when interpreting and comparing findings from prospective and retrospective studies investigating anticancer drug use at end of life.

Chemotherapy was the most frequently assessed treatment type at end of life, since over 90% of studies described its frequency while other anti-cancer treatments were infrequently reported, targeted therapy being the second most reported (7% of articles). About half of the articles described end of life treatment by cancer type: those with high prevalence, such as lung or breast cancer, or with poor prognosis, such as pancreatic cancer, were the most frequently assessed.

The subset of 69 articles on the frequency of end of life anticancer drug use showed that the most investigated end of life periods are the last 2 weeks and the last month of life. The focus on these periods in research on end of life anticancer drug use suggests a recognition of the importance of this period and the need to optimize care during this time to address concerns about the potential harm, reduced quality of life, and increased healthcare costs associated with such interventions. This observation enriches the mounting evidence about aggressive medical intervention at the very end of life period, which has been recognized as inappropriate for terminally ill individuals [36–40]. A less conservative indicator of overtreatment—starting a new anticancer regimen during the last month before death—was rarely reported, although this would allow differentiation between treatments that were started some time earlier from those that were actively initiated later on.

The overall frequency of end of life anticancer drug use showed a wide range for all end of life periods, with the lowest values being close to zero while the highest were 29 and 56% for the last 2 weeks and the last month of life, respectively. We observed differences of varying degrees in the range of frequencies after stratifying for data source, design, size of study population, disease status, treatment characteristics, treatment type, and region. Hospital-based prospective and population-based studies, those with larger sample sizes, that examined previously treated patients and focused on advanced cancer cases, as well as studies involving non-conventional chemotherapy drugs, consistently reported lower frequencies of anticancer drug use across various end of life periods. Our descriptive analysis did not encompass a comprehensive multi-dimensional assessment of individual factors or an evaluation of their interplay. Additional methodological factors may also contribute to treatment frequency, including specific cancer types examined and other considerations in defining the study population. However, our findings are consistent with existing systematic reviews on aggressive end of life care which also demonstrated high variability in the use of indicators of aggressive end of life care and quality of oncological care [16, 17]. In the systematic review conducted by Langton et al. on end of life resource utilization and costs in cancer care, it was concluded that there are several knowledge gaps in this field that need to be addressed through diverse study designs and data sources. For instance, in addition to retrospective decedent designs, studies that include patients who have survived or benefited from aggressive treatments can provide a more comprehensive understanding of all patients living with advanced disease [17]. Our findings present an overview of studies on end of life cancer drug use across different study designs and data sources, including prospective studies, thereby enhancing our knowledge by describing the treatment frequencies observed along these dimensions. According to the review conducted by Adedini et al., the utilization of measures related to aggressive end of life care and oncology quality in the United States is reported to be infrequent and inconsistent. For instance, only 53% of the included studies assessed the use of chemotherapy in the last 14 days of life using validated claims-based measures. Furthermore, initiation of a new chemotherapy regimen in the last month of life was reported in only 6% of the studies reviewed [16]. Our own findings also confirmed the infrequent use of measures for aggressive end of life care, with 66.7% of studies reporting the use of anticancer drugs in last 2 weeks of life and 13.1% reporting the initiation of a new chemotherapy regimen in the last month of life.

### What this study adds

This scoping review presents a comprehensive depiction of research methodologies and outcomes related to end of life anticancer drug use, yielding insights into current treatment frequencies across various end of life periods and revealing the impact of methodological features on observed variance. Interpretation of the described high variability of treatments in the end of life period is challenging since there is a lack of universal quality indicators of end of life cancer management. Studies of these indicators reveal large variations which can be partially explained by differences in healthcare systems and public health policies at the national level, and by heterogeneity in measurement methods and data sources [20, 41, 42]. In some countries such as USA and Australia, doctors are more prone to prescribe anticancer therapies, whereas in others such as Canada, the national healthcare system no longer reimburses the cost beyond a certain number of chemotherapy lines [43]. However, our findings suggest that the frequency of end of life anticancer drug use is not solely governed by geographic factors. Further patient-level factors have also been described as confounders in the patterns of end of life cancer care utilization, like demographic characteristics of patients [44–47], cancer stage [48–50], cancer type [49, 50], survival time [48, 51] and comorbidities [48, 51] beyond the geographical location [41, 51]. Moreover, the variability might reflect differences in patient or family preferences, as well as potential inappropriate decisions made by treating clinicians [52, 53]. The interplay of these factors is complex due to overlapping characteristics, highlighting the evidence gap that remains to better explain the variation in end of life cancer care provision.

### Strengths and weaknesses/limitations of the study

This scoping review has some potential limitations. First, the most recent articles are underrepresented because screening ended in November 2020. However, the COVID-19 pandemic has resulted in disruptions to cancer care, including end of life treatments, as healthcare systems worldwide struggle to balance the needs of cancer patients with the demands of addressing the pandemic [54, 55]. Therefore, we think that the publications for this period do not necessarily fit into the process of changes in end of life care. Second, in spite of the high-sensitivity search strategy, key papers may have been omitted: certain stages of reviews imply a degree of subjectivity, such as selection of studies, and narrative synthesis. However, thanks to the high degree of concordance between the different reviewers during the article screening process, we are confident that the number of potentially missed articles is low. Finally, since our scoping review aimed to assess the frequency of end of life anticancer drug use across comparable publications, we selected studies that were homogeneous in terms of publication dates and cancer types, without considering other potentially relevant methodological factors including patient characteristics, therapy modalities, and disease stages. While the inclusion of these additional factors may have improved the homogeneity of the research, it would have resulted in a significant reduction in the number of pertinent publications and would not have been consistent with the scoping nature of the review.

Despite these potential limitations, our evidence scoping has several strengths. To our knowledge, this is the first evidence scoping review that comprehensively describes the methods applied in primary research studies reporting end of life anticancer drug use. A high number of relevant publications were identified thanks to the extensive and structured literature search and constitute a useful dataset to facilitate further, more specific studies. The research was not restricted to full-text publications, but to ensure exhaustivity we also screened conference abstracts, letters, and editorials. In addition to English, we reviewed publications in Spanish, French, German, Hungarian, Italian, Russian, and Chinese languages reporting primary quantitative data. This scoping review included studies with a minimum of 100 adult patients which reduces the probability of random error arising from low sample sizes.

Future large, multicentre studies would be the most recommended and efficient design to establish the time patterns of end of life anticancer drug use, by assessing the starting date of treatments and analysing data at different time intervals. These future studies on end of life anticancer treatment frequencies should be particularly informative regarding whether treatment is given to patients as part of standard of care or as part of an experimental study, and ideally should collect information about the number of previous treatments and clinical responses, patients’ functional status, their degree of awareness about prognostic and potential side-effects, and eventual palliative care. Moreover, in future studies on end of life anticancer drug use, standardized methodology and study setting are warranted to achieve benchmarkable end of life results.

## Conclusions

Our scoping review provides an exhaustive description of the main characteristics of primary studies that aimed to analyse the treatments administered during the end of life period. Our results show that the proportion of patients receiving cancer-directed treatment at end of life varies widely and was highly prevalent in many publications. However, the large methodological variability across studies poses many challenges in assessing and comparing the extent of overtreatment at the end of life and the importance of its respective determinants. The enormous clinical and social consequences of the excessive use of aggressive treatments in cancer patients with a very poor prognosis require standardized research methods and sound indicators that can be applied widely.

### Supplementary Information

Below is the link to the electronic supplementary material.

Supplementary file1 (DOCX 14 KB)

Supplementary file2 (DOCX 58 KB)

## Data Availability

Not applicable.
