# Supplementary material for: Frequency of anticancer drug use at the end of life: a scoping review
Source: Clin Transl Oncol. 2023 Jun 8;26(1):178–89. doi: 10.1007/s12094-023-03234-1 (PMC10247343; doi:10.1007/s12094-023-03234-1)
Supplement: Supplementary file 1 — Supplementary file1 (DOCX 14 KB) [file 12094_2023_3234_MOESM1_ESM.docx]

# Appendix 1

## Medline Search strategy

(“last day”[tiab] OR “last days”[tiab] OR “last month*”[tiab] OR “until death”[tiab] OR “near death”[tiab] OR “life end”[tiab] OR aggressive*[ti] OR terminal[ti] OR “palliative chemotherapy”[tiab] OR “end of life”[tiab] OR ((day[tiab] OR days[tiab] OR week*[tiab] OR month*[tiab])

AND (life[tiab] OR death[tiab])) OR “end stage”[ti] OR “late stage”[ti] OR “end life”[tiab])

AND (“ctx”[ti] OR “antineoplastic”[tiab] OR “anti cancer”[ti] OR “anticancer”[ti] OR immunotherap*[ti] OR ((cancer[ti] OR malignan*[ti] OR sclc[ti] OR nsclc[ti] OR neoplasm*[ti] OR tumour*[ti] OR tumor*[ti] OR metasta*[ti])

AND (care[ti] OR drug*[ti] OR treatment*[ti] OR therap*[ti] OR hormone*[ti] OR immunotherapy*[ti] OR biologic*[ti])))
