# Supplementary material for: Frequency of anticancer drug use at the end of life: a scoping review
Source: Clin Transl Oncol. 2023 Jun 8;26(1):178–89. doi: 10.1007/s12094-023-03234-1 (PMC10247343; doi:10.1007/s12094-023-03234-1)
Supplement: Supplementary file 2 — Supplementary file2 (DOCX 58 KB) [file 12094_2023_3234_MOESM2_ESM.docx]

# Appendix 2

## **References (N=341)**

1. Aakhus, E; Berman, A T; Crimins, N; Gabriel, P E; Braun, J; Shulman, L N; Evans, T L (2017) Resource utilization and provider variation in the care of patients with stage IV non-small cell lung cancer six months before death. Journal of Clinical Oncology. Conference 35, 8,
2. Abdel-Razeq, H.; Shamieh, O.; Abu-Nasser, M.; Nassar, M.; Samhouri, Y.; Abu-Qayas, B.; Asfour, J.; Jarrah, J.; Abdelrahman, Z.; Ameen, Z.; Al-Hawamdeh, A.; Alomari, M.; Al-Tabba, A.; Al-Rimawi, D.; Hui, D. (2019) Intensity of Cancer Care Near the End of Life at a Tertiary Care Cancer Center in Jordan. Journal of Pain and Symptom Management 57, 6, 1106-1113
3. Abdollah, Firas; Sammon, Jesse D; Majumder, Kaustav; Reznor, Gally; Gandaglia, Giorgio; Sood, Akshay; Hevelone, Nathanael; Kibel, Adam S; Nguyen, Paul L; Choueiri, Toni K; Selvaggi, Kathy J; Menon, Mani; Trinh, Quoc-Dien (2015) Racial Disparities in End-of-Life Care Among Patients With Prostate Cancer: A Population-Based Study. Journal of the National Comprehensive Cancer Network : JNCCN 13, 9, 1131-1138
4. Abdul Monem, Essam; Mehdi, Itrat; Al Bahrani, Bassim Jaffa; Nada, Ayman Mohammad; Al Kharusi, Suad (2014) Utilization of systemic palliative chemotherapy at the end of life: a local experience. JPMA. The Journal of the Pakistan Medical Association 64, 8, 863-868
5. Accordino, M K; Wright, J D; Vasan, S; Neugut, A I; Gross, T; Hillyer, G C; Hershman, D L (2017) Association between survival time with metastatic breast cancer and aggressive end-of-life care. Breast Cancer Research and Treatment 166, 2, 549-558
6. Accordino, M K; Wright, J D; Vasan, S; Neugut, A I; Hillyer, G C; Hershman, D L (2017) Predictors of aggressive end-of-life care in women metastatic breast cancer. Cancer Research. Conference: 39th Annual CTRC AACR San Antonio Breast Cancer Symposium. United States 77, 4,
7. Achkar, T; Jacob, M; Villgran, V; Abberbock, S; Rosenzweig, M Q; Huggins-Puhalla, S L; Brufsky, A; Mathew, A (2015) End-of-life chemotherapy use in metastatic breast cancer. Journal of Clinical Oncology. Conference 33, 15,
8. Adam, H; Hug, S; Bosshard, G (2014) Chemotherapy near the end of life: A retrospective single-centre analysis of patients' charts. BMC Palliative Care 13, 26,
9. Adenis, A; Penel, N; Horn, S; Dominguez, S; Vanhuyse, M; Mirabel, X (2010) Palliative chemotherapy does not improve survival in metastatic esophageal cancer. Oncology 79, 1, 46-54
10. Agarwal, R.; Epstein, A. S.; Saltz, L. B. (2018) Survival outcomes and end of life use of immunotherapy (IO) supplied free of charge for cancer patients. Journal of Clinical Oncology 36, 34,
11. Ahluwalia, S; Ettner, S; Pantoja, P; Lorenz, K; Tisnado, D; Walling, A (2014) Early care planning discussions are associated with less hospital care at the end of life in veterans with advanced cancer. Journal of Pain and Symptom Management 47, 2, 410-411
12. Ahluwalia, S C; Tisnado, D M; Walling, A M; Dy, S M; Asch, S M; Ettner, S L; Kim, B; Pantoja, P; Schreibeis-Baum, H C; Lorenz, K A (2015) Association of Early Patient-Physician Care Planning Discussions and End-of-Life Care Intensity in Advanced Cancer. Journal of Palliative Medicine 18, 10, 834-841
13. Ahmed, A S Z; Santos, F; Dragomir, A; Kassouf, W; Tanguay, S; Aprikian, A (2016) Health-care services utilization during the last 6 months of life among bladder cancer patients who underwent radical cystectomy in Quebec, Canada. Journal of Urology 1, e321-e321
14. Andelkovic, V; Hospital, G C; Coast, G (2013) Palliative chemotherapy during the last month of life. Journal of Clinical Oncology. Conference 31, 15,
15. Andreis, F; Rizzi, A; Rota, L; Meriggi, F; Mazzocchi, M; Zaniboni, A (2011) Chemotherapy use at the end of life. A retrospective single centre experience analysis. Tumori 97, 1, 30-34
16. Ang, E; Newton, L V (2018) Thirty-day mortality after systemic anticancer treatment as a real-world, quality-of-care indicator: the Northland experience. Internal Medicine Journal 48, 4, 403-408
17. Anonymous, (2011) To treat or not to treat cancer patients with chemotherapy at the end of life?. Prescrire International 20, 117, 152-153
18. Anshushaug, M; Aas Gynnild, M; Kvikstad, A; Kaasa, S; Gronberg, B H (2012) A study of cancer therapy during end-of-life care at a regional cancer centre. Palliative Medicine 26, 439-439
19. Asola, R; Huhtala, H; Holli, K (2006) Intensity of diagnostic and treatment activities during the end of life of patients with advanced breast cancer. Breast Cancer Research and Treatment 100, 1, 77-82
20. Assi, T; El Rassy, E; Ibrahim, T; Moussa, T; Tohme, A; El Karak, F; Farhat, F; Faddoul, S; Ghosn, M; Kattan, J (2017) The role of palliative care in the last month of life in elderly cancer patients. Supportive Care in Cancer 25, 2, 599-605
21. Back, A L; Li, Y F; Sales, A E (2005) Impact of palliative care case management on resource use by patients dying of cancer at a veterans affairs medical center. Journal of Palliative Medicine 8, 1, 26-35
22. Baena-Cañada, J. M.; Campini Bermejo, A.; Gámez Casado, S.; Rodríguez Pérez, L.; Quílez Cutillas, A.; Calvete C; enas, J.; Martínez Bautista, M. J.; Benítez Rodríguez, E. (2019) Experiences with Prescribing Large Quantities of Systemic Anticancer Therapy Near Death. Journal of palliative medicine
23. Bahler, C; Signorell, A; Blozik, E; Reich, O (2018) Intensity of treatment in swiss cancer patients at the end-of-life. Cancer Management and Research 10, 481-491
24. Balsalobre-Yago, J.; Martínez-Ortiz, M. J.; Alcázar, M. I. L.; Fuentes, P. C.; Checa-Soriano, A.; Viney, A.; Martínez-Penella, M.; Romero, J. L. A.; Ayala, F. (2018) The impact of inclusion in home palliative program and distance to hospital on chemotherapy near end of life. Journal of Clinical Oncology 36, 15,
25. Bao, Y; Maciejewski, R; Shah, M A; Garrido, M; Maciejewski, P K; Prigerson, H G (2016) Chemotherapy, acute health care events, and hospice use among stage IV pancreatic cancer patients. Journal of Clinical Oncology. Conference 34
26. Bao, Y; Maciejewski, R C; Garrido, M M; Shah, M A; Maciejewski, P K; Prigerson, H G (2018) Chemotherapy Use, End-of-Life Care, and Costs of Care Among Patients Diagnosed With Stage IV Pancreatic Cancer. Journal of Pain and Symptom Management 55, 4, 1113-1121.e3
27. Barbera, L; Paszat, L; Chartier, C (2006) Indicators of poor quality end-of-life cancer care in Ontario. Journal of palliative care 22, 1, 12-17
28. Barbera, L; Paszat, L; Qiu, F (2008) End-of-Life Care in Lung Cancer Patients in Ontario: Aggressiveness of Care in the Population and a Description of Hospital Admissions. Journal of Pain and Symptom Management 35, 3, 267-274
29. Barbera, L; Seow, H; Sutradhar, R; Chu, A; Burge, F; Fassbender, K; McGrail, K; Lawson, B; Liu, Y; Pataky, R; Potapov, A (2015) Quality of end-of-life cancer care in Canada: A retrospective four-province study using administrative health care data. Current Oncology 22, 5, 341-355
30. Bascioni, R; Giorgi, F; Rastelli, F; Di Pietro Paolo, M; Brugni, M; Basirat, F; Mulattieri, S; Giuliodori, L; De Signoribus, G; Silva, R R; Giustini, L (2011) Impact of hospice and palliative home care on chemotherapy use at the end of life (EOL). Journal of Clinical Oncology. Conference: ASCO Annual Meeting 29, 15,
31. Beaussant, Yvan; Daguindau, Etienne; Chauchet, Adrien; Rochigneux, Philippe; Tournigand, Christophe; Aubry, Regis; Morin, Lucas (2018) Hospital end-of-life care in haematological malignancies. BMJ supportive & palliative care 8, 3, 314-324
32. Beck, A. C. (2019) Making treatment decisions at end of life in a comprehensive cancer center. Journal of Clinical Oncology 51
33. Bolzonello, S; Gerratana, L; Poletto, E; Bonotto, M; Fanotto, V; Cinausero, M; Bernardis, A; Pisa, F E; Fasola, G (2015) Treatment during the last month of life in advanced cancer patients. Journal of Clinical Oncology. Conference 33, 15,
34. Bouleuc, C; Czapiuk, G; Laurence, V; Guilhaume, M N; Gridel, G; Copel, L (2011) Impact of time without chemotherapy before death in end of life care. Supportive Care in Cancer 1, S175-S175
35. Bradley, C J; Yabroff, K R; Mariotto, A B; Zeruto, C; Tran, Q; Warren, J L (2017) Antineoplastic treatment of advanced-stage non-small-cell lung cancer: Treatment, survival, and spending (2000 to 2011). Journal of Clinical Oncology 35, 5, 529-535
36. Braga, S; Miranda, A; Fonseca, R; Passos-Coelho, J L; Fernandes, A; Costa, J D; Moreira, A (2007) The aggressiveness of cancer care in the last three months of life: A retrospective single centre analysis. Psycho-Oncology 16, 9, 863-868
37. Bremner, K. E. (2020) Patterns of Care and Costs for Older Patients With Colorectal Cancer at the End of Life: Descriptive Study of the United States and Canada. JCO Oncol Pract e1-e18
38. Caraceni, A; Zecca, E; Grecchi, S; Vitali, M; Ricchini, F; Pigni, A; Brunelli, C; Garassino, M (2016) Quality of care provided within a model for early palliative care integration in advanced lung cancer patients. Palliative Medicine 30, NP137-NP137
39. Cassel, B; Skoro, N; Kerr, K; Shickle, L; Coyne, P J; Del Fabbro, E (2012) Retrospective assessment of quality of cancer care in last 6 months of life. Journal of Clinical Oncology. Conference: ASCO's Quality Care Symposium 30, 34,
40. Chan, K (2012) Aggressiveness of cancer-care in lung cancer patients near the end-of-life in an oncology center in Hong Kong. Journal of Pain Management 5, 1, 71-82
41. Chan, T; Ewachiw, B; Huang, P; Frendak, L; Waldfogel, J; Burdalski, C; Feliciano, J (2018) Prescribing Patterns of Physicians and Financial Implications for Lung Cancer Treatment at the End of Life. Journal of Thoracic Oncology 13, S818-S819
42. Chang, T S; Su, Y C; Lee, C C (2015) Determinants for aggressive end-of-life care for oral cancer patients: A population-based study in an Asian country. Medicine 94, 4,
43. Chanprasertpinyo, W; Semsarn, S; Tangsujaritvijit, V; Ngamphaiboon, N; Reungwetwattana, T; Chaiviboontham, S; Konmun, J; Sachdev, V; Chansriwong, P (2017) Effect of early palliative care on aggressiveness of cancer care near end of life in lung cancer patient. Journal of Thoracic Oncology 12, S1774-S1775
44. Check, D K; Rosenstein, D L; Dusetzina, S B (2016) Early supportive medication use and end-of-life care among Medicare beneficiaries with advanced breast cancer. Supportive Care in Cancer 24, 8, 3463-3472
45. Cheng, Benjamin Hon Wai; Sham, Michael Mau Kwong; Chan, Kwok Ying; Li, Cho Wing; Au, Ho Yan (2015) Intensive palliative care for patients with hematological cancer dying in hospice: analysis of the level of medical care in the final week of life. The American journal of hospice & palliative care 32, 2, 221-225
46. Cheung, M C; Earle, C C; Rangrej, J; Ho, T H; Liu, N; Barbera, L; Saskin, R; Porter, J; Seung, S J; Mittmann, N (2015) Impact of aggressive management and palliative care on cancer costs in the final month of life. Cancer 121, 18, 3307-3315
47. Chew, L; Chan, Z Y; Phebe, S E H (2014) Evaluating factors associated with life expectancy less than 3 months among elderly patients receiving palliative chemotherapy. Journal of Oncology Pharmacy Practice 1, 23-23
48. Chiang, Jui-Kun; Kao, Yee-Hsin; Lai, Ning-Sheng (2015) The Impact of Hospice Care on Survival and Healthcare Costs for Patients with Lung Cancer: A National Longitudinal Population-Based Study in Taiwan. PLoS ONE 10, 9, e0138773-e0138773
49. Chiang, J K; Hsu, C S; Lin, C W; Kao, Y H (2018) End-of-Life Care for Patients with Advanced Ovarian Cancer Is Aggressive Despite Hospice Intervention: A Population-Based Study. International Journal of Gynecological Cancer 28, 6, 1183-1190
50. Chiang, J. K. (2019) Association between palliative care and end-of-Life care for patients with hematological malignancies: A population-based study. Medicine (Baltimore) e17395
51. Choi, Y; Keam, B; Kim, T M; Lee, S H; Kim, D W; Heo, D S (2014) Cancer care near the end of life (EOL) in the era of molecular-targeted agents: Changes of trend during 10 years at single institution. Journal of Clinical Oncology. Conference 32, 15,
52. Choi, Younak; Keam, Bhumsuk; Kim, Tae Min; Lee, Se-Hoon; Kim, Dong-Wan; Heo, Dae Seog (2015) Cancer Treatment near the End-of-Life Becomes More Aggressive: Changes in Trend during 10 Years at a Single Institute. Cancer research and treatment : official journal of Korean Cancer Association 47, 4, 555-563
53. Choi, C. (2019) Evaluation of Palliative Treatments in Unresectable Pancreatic Cancer Patients. HPB S413
54. Cinausero, M; Gerratana, L; De Carlo, E; Iacono, D; Bonotto, M; Fanotto, V; Buoro, V; Basile, D; Vitale, M G; Rihawi, K; Fasola, G; Puglisi, F (2018) Determinants of Last-line Treatment in Metastatic Breast Cancer. Clinical Breast Cancer 18, 3, 205-213
55. Colla, C H; Morden, N E; Skinner, J S; Hoverman, J R; Meara, E (2012) Impact of payment reform on chemotherapy at the end of life. American Journal of Managed Care 18, 5, e200-e208
56. Collins, A; Burchell, J L; Sundararajan, V; Krishnasamy, M; Mileshkin, L; McLachlan, S A; Le, B; Hudson, P; Currow, D; Millar, J; Philip, J (2016) Integration of palliative care for patients with metastatic breast cancer: Have we achieved quality end-of-life care?. Palliative Medicine 30, NP177-NP178
57. Colombet, I; Montheil, V; Durand, J P; Gillaizeau, F; Niarra, R; Jaeger, C; Alexandre, J; Goldwasser, F; Vinant, P (2012) Effect of integrated palliative care on the quality of end-of-life care: retrospective analysis of 521 cancer patients. BMJ supportive & palliative care 2, 3, 239-247
58. Colombet, I.; Bouleuc, C.; Piolot, A.; Vilfaillot, A.; Jaulmes, H.; Voisin-Saltiel, S.; Goldwasser, F.; Vinant, P. (2019) Multicentre analysis of intensity of care at the end-of-life in patients with advanced cancer, combining health administrative data with hospital records: variations in practice call for routine quality evaluation. BMC Palliative Care 18, 1,
59. Cottini, S; Bria, E; Sperduti, I; Tortora, G (2015) Appropriateness and cost analyses of anticancer treatments (AT) in the last 3 months of life: A retrospective single center cohort study. Annals of Oncology. Conference: 17th National Congress of Medical Oncology. Rome Italy. Conference Publication: 26
60. Dallara, A; Carver, A; Diamond, E (2014) Aggressive medical care at the end of life (EOL) in glioblastoma (GBM): The 5-year memorial sloan-kettering cancer center (MSKCC) experience. Neurology. Conference: 66th American Academy of Neurology Annual Meeting, AAN 82, 10,
61. Datta, S; Kelly, V; Maguire, J (2013) Palliative chemotherapy with fractionated carboplatin and vinorelbine for elderly and poor performance status patients with NSCLC. Lung Cancer 1, S13-S13
62. de Man, Y.; Atsma, F.; Oosterveld-Vlug, M. G.; Brom, L.; Onwuteaka-Philipsen, B. D.; Westert, G. P.; Groenewoud, A. S. (2019) The Intensity of Hospital Care Utilization by Dutch Patients With Lung or Colorectal Cancer in their Final Months of Life. Cancer Control 26, 1,
63. de Oliveira Valentino, T. C. (2020) Impact of Palliative Care on Quality of End-of-Life Care Among Brazilian Patients With Advanced Cancers. J Pain Symptom Manage 39-48
64. De Schreye, R; Smets, T; Annemans, L; Deliens, L; Gielen, B; De Gendt, C; Cohen, J (2017) Applying quality indicators for administrative databases to evaluate end-of-life care for cancer patients in Belgium. Health Affairs 36, 7, 1234-1243
65. Debled, M; Madranges, N; Mertens, C; Durand, M; Brouste, V; Brain, E; Mauriac, L (2011) First-line chemotherapy for metastatic breast cancer in patients >=75 years: A retrospective single-centre analysis. Critical Reviews in Oncology/Hematology 80, 1, 171-179
66. Diaz, L; Jeanpierre, M; Berod, T (2016) Chemotherapy orders near the end of life: A retrospective study in a French Nonteaching Hospital. Pharmacien Hospitalier et Clinicien 51, 1, e1-e5
67. Do, K; Sadeghi, S; Matsuura, P; Lynch, G; Barzi, A (2017) Characteristics of patients (pts) receiving end of life treatment (EOLT) at an NCIDesignated Cancer Center. Journal of Clinical Oncology. Conference 35, 15,
68. Dobrila-Dintinjana, R; Redzovic, A; Peric, J; Petranovic, D (2013) The approaches in the care for terminal cancer patients in radiotherapy and oncology clinic, Rijeka University Hospital Center. Collegium Antropologicum 37, 287-290
69. Dougherty, D W; Kadlubek, P; Pham, T; Earle, C; Malin, J; Breathwaite, L; Jacobson, J O (2013) Opportunities for improved end-of-life (EOL) care for adult patients with advanced cancer: Results of a longitudinal assessment of care provided by Quality Oncology Practice Initiative (QOPI) participants. Journal of Clinical Oncology. Conference: ASCO's Quality Care Symposium 31, 31,
70. Douma, G; Fransen, H P; Venmans, B J W; Aarts, M J (2017) End of life treatment of metastatic lung cancer patients in the Netherlands. European Respiratory Journal. Conference: European Respiratory Society International Congress, ERS 50
71. Douma, G; Fransen, H; Venmans, B; Aarts, M (2018) End of life treatment of metastatic lung cancer patients in The Netherlands, a population-based study. Palliative Medicine 32, 38-38
72. Dudevich, A; Chen, A; Gula, C; Fagbemi, J (2014) End-of-life hospital care for cancer patients: an update. Healthcare quarterly (Toronto, Ont.) 17, 3, 8-10
73. Earle, C C; Neville, B A; Landrum, M B; Ayanian, J Z; Block, S D; Weeks, J C (2004) Trends in the aggressiveness of cancer care near the end of life. Journal of Clinical Oncology 22, 2, 315-321
74. Earle, C C; Neville, B A; Landrum, M B; Souza, J M; Weeks, J C; Block, S D; Grunfeld, E; Ayanian, J Z (2005) Evaluating claims-based indicators of the intensity of end-of-life cancer care. International Journal for Quality in Health Care 17, 6, 505-509
75. Earle, C C; Landrum, M B; Souza, J M; Neville, B A; Weeks, J C; Ayanian, J Z (2008) Aggressiveness of cancer care near the end of life: Is it a quality-of-care issue?. Journal of Clinical Oncology 26, 23, 3860-3866
76. Ebert Moltara, M; Mesti, T; Mrsnik, M; Benedik, J; Ivanetic, M; Cervek, M; Rajer, M; Zavratnik, B; Unk, M; Tonkli, A; Ravnik, M; Horvat, M; Gregoric, B; Pelipenko, K; Zakotnik, B; Cervek, J (2012) Adverse events of chemotherapy near the end of life. Palliative Medicine 26, 612-613
77. Ebert Moltara, M; Pahole Golicnik, J; Vidali, G; Saje, A; Ivanetic, M; Zist, A; Cervek, J (2014) Use of chemotherapy and target therapy in the last weeks of life. Palliative Medicine 28, 757-758
78. Edman Kessler, L. (2019) Administration of chemotherapy for metastatic breast cancer near the end of life: A population registry study. Annals of Oncology v136
79. Edman Kessler, L. (2020) Chemotherapy use near the end-of-life in patients with metastatic breast cancer. Breast Cancer Res Treat 645-651
80. Edna, Tom- H; Jullumstro, Eivind; Lydersen, Stian (2008) Chemotherapy for nonresectable colorectal cancer at a center during 25 years. Hepato-Gastroenterology 55, 88, 2049-2053
81. Egan, P. C. (2020) End-of-life care quality outcomes among Medicare beneficiaries with hematologic malignancies. Blood Adv 3606-3614
82. Emanuel, E J; Young-Xu, Y; Levinsky, N G; Gazelle, G; Saynina, O; Ash, A S (2003) Chemotherapy Use among Medicare Beneficiaries at the End of Life. Annals of Internal Medicine 138, 8, 639-643+I44
83. Ersek, M; Miller, S C; Wagner, T H; Thorpe, J M; Smith, D; Levy, C R; Gidwani, R; Faricy-Anderson, K; Lorenz, K A; Kinosian, B; Mor, V (2017) Association between aggressive care and bereaved families' evaluation of end-of-life care for veterans with non-small cell lung cancer who died in Veterans Affairs facilities. Cancer 123, 16, 3186-3194
84. Falchook, A D; Dusetzina, S B; Tian, F; Basak, R; Selvam, N; Chen, R C (2017) Aggressive End-of-Life Care for Metastatic Cancer Patients Younger Than Age 65 Years. Journal of the National Cancer Institute 109, 9,
85. Faluyi, O; Connor, J; Chatterjee, B; Ikin, C; Wong, H; Palmer, D (2016) Advanced pancreatic adenocarcinoma outcomes with transition from devolved to centralised care in a UK regional cancer centre. Annals of Oncology. Conference: 41st European Society for Medical Oncology Congress, ESMO 27
86. Fang, P.; Jagsi, R.; He, W.; Lei, X.; Campbell, E. G.; Giordano, S. H.; Smith, G. L. (2019) Rising and Falling Trends in the Use of Chemotherapy and Targeted Therapy Near the End of Life in Older Patients With Cancer. Journal of clinical oncology : official journal of the American Society of Clinical Oncology JCO1802067
87. Feuerlein, K; De Dosso, S; Castillo, F O; Frigerio, M; Ghielmini, M; Saletti, P (2011) How much chemotherapy are patients with advanced pancreatic cancer receiving at the end of life?. European Journal of Cancer 1, S468-S468
88. Fitzgibbon, E; Boucher, C (2010) Is visiting the Emergency Department indicative of poor quality end of life cancer care?: Prevalence of 'do not resucitate' orders and advance care directives among cancer patients who attended the Emergency Department at the end of life. Supportive Care in Cancer 3, S162-S162
89. Fond, G. (2020) End-of-Life Care Among Patients With Bipolar Disorder and Cancer: A Nationwide Cohort Study. Psychosom Med 722-732
90. Frigeri, M; De Dosso, S; Castillo-Fernandez, O; Feuerlein, K; Neuenschwander, H; Saletti, P (2013) Chemotherapy in patients with advanced pancreatic cancer: Too close to death?. Supportive Care in Cancer 21, 1, 157-163
91. Fuchs, Fabiola; Robausch, Martin (2018) [End-of-life therapy for patients dying with cancer: a retrospective database study]. Krebserkrankungen - Therapie am Lebensende: eine retrospektive Datenanalyse. 168, 13, 344-349
92. Fujisawa, D; Temel, J S; Traeger, L; Greer, J A; Lennes, I T; Mimura, M; Pirl, W F (2015) Psychological factors at early stage of treatment as predictors of receiving chemotherapy at the end of life. Psycho-Oncology 24, 12, 1731-1737
93. Futagami, Masayuki; Yokoyama, Yoshihito; Sato, Tetsumi; Hirota, Kazuyoshi; Shimada, Muneaki; Miyagi, Etsuko; Suzuki, Nao; Fujimura, Masaki (2016) Palliative Care for Patients with Gynecologic Cancer in Japan: A Japan Society of Gynecologic Palliative Medicine (JSGPM) Survey. Asian Pacific journal of cancer prevention : APJCP 17, 10, 4637-4642
94. Gallais Serezal, I; Beaussant, Y; Rochigneux, P; Tournigand, C; Aubry, R; Lindelof, B; Morin, L (2016) End-of-life care for hospitalized patients with metastatic melanoma in France: A nationwide, register-based study. British Journal of Dermatology.
95. García Martín, E.; Escudero Vilaplana, V.; Collado Borrell, R.; González-Haba Peña, E.; Marzal Alfaro, B.; Fox, B.; Hoyo Muñoz, Á; Martínez Ortega, P. A.; Amor García, M. Á; Herranz Alonso, A.; Sanjurjo Saéz, M. (2019) Assessment of aggressive care in oncology patients at the end of life in clinical practice. European Journal of Hospital Pharmacy 26, A120-A121
96. Geerse, O P; Hoekstra-Weebers, J E H M; Stokroos, M H; Burgerhof, J G M; Groen, H J M; Kerstjens, H A M; Hiltermann, T J N (2017) Structural distress screening and supportive care for patients with lung cancer on systemic therapy: A randomised controlled trial. European Journal of Cancer 72, 37-45
97. Gibson, A. J. W.; Li, H.; D'Silva, A.; Elegbede, A. A.; Tudor, R. A.; Otsuka, S.; Bebb, D. G.; Cheung, W. Y. (2019) Factors associated with early mortality in non-small cell lung cancer patients following systemic anti-cancer therapy: A 10 year population-based study. Lung Cancer 134, 141-146
98. Gilbar, Peter J; McPherson, Ian; Aisthorpe, Genevieve G; Kondalsamy-Chennakes, Srinivas (2019) Systemic anticancer therapy in the last 30 days of life: Retrospective audit from an Australian Regional Cancer Centre. Journal of oncology pharmacy practice : official publication of the International Society of Oncology Pharmacy Practitioners 1078155217752077-1078155217752077
99. Gofton, T E; Graber, J; Carver, A (2012) Identifying the palliative care needs of patients living with cerebral tumors and metastases: A retrospective analysis. Journal of Neuro-Oncology 108, 3, 527-534
100. Goldwasser, F; Vinant, P; Huillard, O; Morin, L (2016) Goals and aggressiveness of care in metastatic lung cancer. Journal of Clinical Oncology. Conference 34
101. Goncalves, J F; Goyanes, C (2008) Use of chemotherapy at the end of life in a Portuguese oncology center. Supportive Care in Cancer 16, 4, 321-327
102. Gonsalves, W I; Tashi, T; Krishnamurthy, J; Davies, T; Ortman, S; Thota, R; Aldoss, I; Ganta, A; Kalaiah, M; Didwaniya, N; Eberle, C; Ganti, A K; Silberstein, P T; Subbiah, S (2011) Effect of palliative care services on the aggressiveness of end-of-life care in the veteran's affairs cancer population. Journal of Palliative Medicine 14, 11, 1231-1235
103. Goody, R B; Choong, E S; Calderwood, J; Law, S J M; Mazdai, G; Hanna, G G; McAleer, J J A (2009) Mortality within 30 days for patients older than 70 years receiving chemotherapy: A single-institution retrospective analysis. Journal of the Hong Kong College of Radiologists 12, 3, 95-102
104. Green, J B; Shapiro, M F; Ettner, S; Malin, J; Wong, M D (2011) Chemotherapy use in lung cancer at the end of life: Predictors and variation in use. Journal of General Internal Medicine 1, S94-S95
105. Green, J B; Shapiro, M F; Ettner, S L; Malin, J; Ang, A; Wong, M D (2017) Patients receiving care for advanced non-small cell lung cancer in small, independent oncology practices are more likely to receive chemotherapy in the last 30 days of life. American Journal of Managed Care 23, 4,
106. Grendarova, P; Sinnarajah, A; Trotter, T; Card, C; Wu, J S Y (2015) Variations in intensity of end-of-life cancer therapy by cancer type at a Canadian tertiary cancer centre between 2003 and 2010. Supportive Care in Cancer 23, 10, 3059-3067
107. Griffiths, R W; Zee, Y K; Evans, S; Mitchell, C L; Kumaran, G C; Welch, R S; Jayson, G C; Clamp, A R; Hasan, J (2011) Outcomes after multiple lines of chemotherapy for platinum-resistant epithelial cancers of the ovary, peritoneum, and fallopian tube. International Journal of Gynecological Cancer 21, 1, 58-65
108. Guadagnolo, B Ashleigh; Liao, Kai-Ping; Giordano, Sharon H; Elting, Linda S; Shih, Ya-Chen T (2015) Variation in Intensity and Costs of Care by Payer and Race for Patients Dying of Cancer in Texas: An Analysis of Registry-linked Medicaid, Medicare, and Dually Eligible Claims Data. Medical care 53, 7, 591-598
109. Gynnild, M A; Anshushaug, M; Kaasa, S; Kvikstad, A; Gronberg, B H (2012) Palliative cancer therapy during end of life at a regional cancer center in Norway in 2005 and 2009. Journal of Clinical Oncology. Conference 30, 15,
110. Hammerman, A; Greenberg-Dotan, S; Battat, E; Bitterman, H; Ariad, S (2014) Chemotherapy use in lung cancer patients during last three months of life: Current practice in a large Israeli health care organization. Journal of Clinical Oncology. Conference 32, 15,
111. Hashimoto, Kenji; Yonemori, Kan; Katsumata, Noriyuki; Hotchi, Marika; Kouno, Tsutomu; Shimizu, Chikako; Tamura, Kenji; Ando, Masashi; Takeuchi, Masahiro; Fujiwara, Yasuhiro (2009) Factors that affect the duration of the interval between the completion of palliative chemotherapy and death. The oncologist 14, 7, 752-759
112. Hassan, A; Allam, A; Mohsen, H (2015) Trends in the aggressiveness of end-of-life cancer care in the State of Qatar. Supportive Care in Cancer 1, S283-S283
113. Hassan, A; Elazzazy, S; Haddad, P (2016) End of life care for haematologic malignancies: A retrospective cohort study from the state of qatar. Supportive Care in Cancer 25, S227-S227
114. Haukland, (2020) Adverse events in deceased hospitalised cancer patients as a measure of quality and safety in end-of-life cancer care. BMC Palliative Care
115. Herrel, L; Kaye, D; Min, A; Ellimoottil, C; Dupree, J M; Miller, D (2018) Accountable care organizations and the cost and quality of urological cancer care at the end of life. Journal of Urology 199, e1019-e1019
116. Hiramoto, S.; Tamaki, T.; Nagashima, K.; Hori, T.; Kikuchi, A.; Yoshioka, A.; Inoue, A. (2018) Prognostic factors in patients who received end-of-life chemotherapy for advanced cancer. International Journal of Clinical Oncology 24, 4, 454-459
117. Hirvonen, O. M. (2019) Assessing the utilization of the decision to implement a palliative goal for the treatment of cancer patients during the last year of life at Helsinki University Hospital: a historic cohort study. Acta Oncol 1699-1705
118. Ho, T H; Barbera, L; Saskin, R; Lu, H; Neville, B A; Earle, C C (2011) Trends in the aggressiveness of end-of-life cancer care in the universal health care system of Ontario, Canada. Journal of Clinical Oncology 29, 12, 1587-1591
119. Hoberg, J; Alt-Epping, B; Freier, W; Griesinger, F; Strasser, F; Nauck, F (2010) Do oncology and palliative care institutions respond differently to the needs of patients with lung cancer in their last month of life? Preliminary results from a multicenter survey. Palliative Medicine 1, S100-S100
120. Hong, Ji Hyung; Rho, Sang-Young; Hong, Young Seon (2013) Trends in the Aggressiveness of End-of-Life Care for Advanced Stomach Cancer Patients. Cancer research and treatment : official journal of Korean Cancer Association 45, 4, 270-275
121. Hu, W; Yasui, Y; White, J; Winget, M (2014) Aggressiveness of end-of-life care for patients with colorectal cancer in Salberta, Canada: 2006-2009. Journal of Pain and Symptom Management 47, 2, 231-244
122. Hu, J; Aprikian, A; Vanhuyse, M; Dragomir, A (2018) Use of Cancer Drugs in the End-of-Life in Men Dying of Castration-Resistant Prostate Cancer: Population-Based Study. Value in Health 21, S56-S56
123. Hu, J.; Aprikian, A. G.; Vanhuyse, M.; Dragomir, A. (2019) Cancer Drug Use in the Last Month of Life in Men With Castration-Resistant Prostate Cancer. Journal of oncology practice 15, 6, e510-e519
124. Huang, H K; Wang, Y W; Hsieh, J G; Hsieh, C J (2017) Disparity of end-of-life care in cancer patients with and without schizophrenia: A nationwide population-based cohort study. Schizophrenia Research 195, 434-440
125. Hugar, L. A.; Lopa, S. H.; Yabes, J.; Hale, N.; Turner, R.; Fam, M. M.; Macleod, L. C.; Davies, B. J.; Jacobs, B. L. (2018) Aggressive end-of-life care in Medicare bbeneficiaries dying with bladder cancer. Canadian Urological Association Journal 12, 9, S218-S219
126. Huillard, O; Goldwasser, F; Morin, L (2016) Aggressiveness of care at the end of life in patients with localized and advanced bladder cancer. Journal of Clinical Oncology. Conference 34
127. Jang, R W; Krzyzanowska, M K; Zimmermann, C; Alibhai, S (2012) The impact of palliative care on the aggressiveness of end-of-life cancer care in patients with advanced pancreatic cancer. Annals of Oncology 9, ix464-ix465
128. Jang, R W J; Krzyzanowska, M K; Zimmermann, C; Taback, N; Alibhai, S M H (2013) Intensity of palliative care and its impact on the aggressiveness of end-of-life care in patients with advanced pancreatic cancer. Journal of Clinical Oncology. Conference 31, 15,
129. Jang, T K; Kim, D Y; Lee, S W; Park, J Y; Suh, D S; Kim, J H; Kim, Y M; Kim, Y T; Nam, J H (2018) Trends in treatment during the last stages of life in end-stage gynecologic cancer patients who received active palliative chemotherapy: a comparative analysis of 10-year data in a single institution. BMC Palliative Care 17, 1, 1-8
130. Jessop, S; Sillah, T; Jeffs, Y; Aslam, S; Bulusu, V (2016) 30 day mortality following systemic anti-cancer treatment (SACT) in lung cancer: Experience from a cancer unit. Lung Cancer 91, S30-S30
131. Jho, H. J. (2020) Changes of End of Life Practices for Cancer Patients and Their Association with Hospice Palliative Care Referral over 2009-2014: A Single Institution Study. Cancer Res Treat 419-425
132. Jung, Daeun; Hwang, Sunjin; You, Hyun Jung; Lee, Jungkwon (2012) The realities and associated factors of palliative chemotherapy near the end of life in the patients enrolled in palliative care unit. Korean journal of family medicine 33, 1, 44-50
133. Kalisiak, A; Glenn, L A; Weinmeister, M (2014) Extended impact of an embedded palliative care program in a community cancer center. Journal of Clinical Oncology. Conference: Palliative Care in Oncology Symposium 32, 31,
134. Kao, S; Shafiq, J; Vardy, J; Adams, D (2009) Use of chemotherapy at end of life in oncology patients. Annals of Oncology 20, 9, 1555-1559
135. Kao, S C H; Clarke, S; Clarke, C; Corte, P; Van Zandwijk, N; Vardy, J (2011) End of life care for malignant pleural mesothelioma (MPM) patients in Australia. Asia-Pacific Journal of Clinical Oncology 4, 136-136
136. Kao, S C; Van Zandwijk, N; Corte, P; Clarke, C; Clarke, S; Vardy, J (2013) Use of cancer therapy at the end of life in patients with malignant pleural mesothelioma. Supportive Care in Cancer 21, 7, 1879-1884
137. Karanth, S; Rajan, S S; Sharma, G; Yamal, J M; Morgan, R O (2018) Racial-Ethnic Disparities in End-of-Life Care Quality among Lung Cancer Patients: A SEER-Medicare-Based Study. Journal of Thoracic Oncology 13, 8, 1083-1093
138. Karim, S M; Zekri, J M; Abdelghany, E M; Rizvi, A; Al-Gahmi, A; Munsoor, H; Ahmad, I; Dada, R; Ansari, M I; Kerr, I G (2013) Impact of palliative care on end-of-life management of cancer patients dying in the hospital. Journal of Clinical Oncology. Conference 31, 15,
139. Karim, S M; Zekri, J; Abdelghany, E; Dada, R; Munsoor, H; Ahmad, I (2015) Time from last chemotherapy to death and its correlation with the end of life care in a referral hospital. Indian Journal of Medical and Paediatric Oncology 36, 1, 55-59
140. Keam, Bhumsuk; Oh, Do-Youn; Lee, Se-Hoon; Kim, Dong-Wan; Kim, Mi Ra; Im, Seock-Ah; Kim, Tae-You; Bang, Yung-Jue; Heo, Dae Seog (2008) Aggressiveness of cancer-care near the end-of-life in Korea. Japanese journal of clinical oncology 38, 5, 381-386
141. Keating, N L; Landrum, M B; Lamont, E B; Earle, C C; Bozeman, S R; McNeil, B J (2010) End-of-life care for older cancer patients in the veterans health administration versus the private sector. Cancer 116, 15, 3732-3739
142. Keating, N. L.; O'Malley, A. J.; Onnela, J. P.; Gray, S. W.; on, B. E. (2019) Influence of Peer Physicians on Intensity of End-of-Life Care for Cancer Decedents. Medical Care 57, 6, 468-474
143. Kehl, K. L. (2018) Hospitalization by cytotoxic chemotherapy regimen among older women with stage IV breast cancer. Cancer 4685-4691
144. Kempf, E; Morin, L (2016) Use of treatments of questionable benefit in hospitalized patients with metastatic gastric or esophageal cancer near the end of life. A country-wide, register-based study. Annals of Oncology. Conference: 41st European Society for Medical Oncology Congress, ESMO 27
145. Kempf, E; Tournigand, C; Rochigneux, P; Aubry, R; Morin, L (2017) Discrepancies in the use of chemotherapy and artificial nutrition near the end of life for hospitalised patients with metastatic gastric or oesophageal cancer. A countrywide, register-based study. European Journal of Cancer 79, 31-40
146. Kerba, M; Sinnarajah, A; Rose, M S; Nicholson, L; Wheler, B; Enns, B (2015) End-of-life cancer care: Health service delivery in the last 12 months of life in Calgary, Alberta, Canada. Journal of Clinical Oncology. Conference 33, 29,
147. Khaki, A. R. (2020) Comparison of Health Care Utilization at the End of Life Among Patients With Cancer in Alberta, Canada, Versus Washington State. JCO Oncol Pract Op2000217
148. Kihara, R.; Ishiguri, Y.; Ueda, N.; Asai, Y.; Odagiri, T.; Watamoto, K.; Watanabe, H. (2018) Effect of speciallist palliative care service on end-of life care in patients with hematologic malignancies. Blood 132,
149. Kim, H.; Keating, N. L.; Perloff, J. N.; Hodgkin, D.; Liu, X.; Bishop, C. E. (2019) Aggressive Care near the End of Life for Cancer Patients in Medicare Accountable Care Organizations. Journal of the American Geriatrics Society 67, 5, 961-968
150. Kimura, M; Kawachi, S; Go, M; Iwai, M; Usami, E; Teramachi, H; Yoshimura, T (2019) Effect of the timing of discontinuation of last-line chemotherapy on patient prognosis in advanced and recurrent gastric cancer. Molecular and Clinical Oncology 10, 1, 173-179
151. King, J D; Retseck, J; Eickhoff, J C; Hoang, T; Traynor, A M; Campbell, T C (2013) Integrated oncopalliative care versus standard care for patients with metastatic lung cancer: A single institution retrospective review. Journal of Clinical Oncology. Conference 31, 15,
152. Kok, P S; Chan, H; Chao, C; Descallar, J; Bray, V; Tognela, A; Yip, P Y (2015) Timing of palliative care referral and its impact on receiving aggressive end of life care in patients with metastatic non-small cell lung cancer (NSCLC) in Southwest Sydney. Annals of Oncology 9, ix111-ix111
153. Kolodziej, M; Hoverman, J R; Garey, J S; Espirito, J; Sheth, S; Ginsburg, A; Neubauer, M A; Patt, D; Brooks, B; White, C; Sitarik, M; Anderson, R; Beveridge, R (2011) Benchmarks for value in cancer care: An analysis of a large commercial population. Journal of Oncology Practice 7, 5, 301-306
154. Koroukian, S M; Schiltz, N K; Warner, D F; Given, C W; Schluchter, M; Owusu, C; Berger, N A (2017) Social determinants, multimorbidity, and patterns of end-of-life care in older adults dying from cancer. Journal of Geriatric Oncology 8, 2, 117-124
155. Kraut, J; Gippetti, J; Peterson, D; Agarwala, V; Revol, C; Fessele, K L; Abernethy, A P (2017) Chemotherapy use near end of life (EOL): Measuring real world benchmarks. Journal of Clinical Oncology. Conference 35, 8,
156. Kuppen, M; Westgeest, H; Van Den Eertwegh, A; Gerritsen, W; Uyl-De Groot, C (2017) Use of new therapies and hospital admission near the end of life in castration resistant prostate cancer (CRPC) in the castration resistant prostate cancer registry (CAPRI) in the Netherlands. Value in Health 20, A400-A400
157. Kvale, E A; Rocque, G; Bevis, K S; Acemgil, A; Taylor, R A; Demark-Wahnefried, W; Kenzik, K; Li, Y; Meneses, K; Martin, M; Fouad, M N; Pisu, M; Partridge, E E (2015) Trends in health care utilization, cost, and aggressive care at end of life among older cancer patients in the Deep South. Journal of Clinical Oncology. Conference 33, 29,
158. Labrant, L; Rimel, B J; Walsh, C; Li, A; Karlan, B; Cass, I (2013) Too much, too late: Aggressive care in women with recurrent gynecologic malignancies and the ttiming of end-of life discussions. Gynecologic Oncology 131, 255-255
159. Lafuma, A.; Cotté, F. E.; Le Tourneau, C.; Emery, C.; Gaudin, A. F.; Torreton, E.; Gourmelen, J.; Bonastre, J. (2019) Economic burden of chemotherapy-treated recurrent and/or metastatic squamous cell carcinoma of the head and neck in France: real-world data from the permanent sample of national health insurance beneficiaries. Journal of Medical Economics 22, 7, 698-705
160. Lammers, A.; Slatore, C. G.; Fromme, E. K.; Vranas, K. C.; Sullivan, D. R. (2019) Association of Early Palliative Care With Chemotherapy Intensity in Patients With Advanced Stage Lung Cancer: A National Cohort Study. Journal of Thoracic Oncology 14, 2, 176-183
161. Ledoux, Mathilde; Rhondali, Wadih; Lafumas, Veronique; Berthiller, Julien; Teissere, Marion; Piegay, Celine; Couray-Targe, Sandrine; Schott, Anne-Marie; Bruera, Eduardo; Filbet, Marilene (2015) Palliative care referral and associated outcomes among patients with cancer in the last 2 weeks of life. BMJ supportive & palliative care
162. Ledoux, M; Tricou, C; Roux, M; Dreano-Hartz, S; Ruer, M; Filbet, M (2018) Cancer Patients Dying in the Intensive Care Units and Access to Palliative Care. Journal of Palliative Medicine 21, 5, 689-693
163. Lee, S W; Kim, S Y; Baek, J Y; Shim, E K; Kim, H M; Ku, J Y; Nam, E J; Jho, H J; Chang, Y J (2015) Impact of outpatient palliative care on aggressiveness of end-of-life care in patients with metastatic colorectal cancer. European Journal of Cancer 3, S208-S209
164. Lee, Si Won; Jho, Hyun Jung; Baek, Ji Yeon; Shim, Eun Kyung; Kim, Hyun Mi; Ku, Ji Yeon; Nam, Eun Jung; Chang, Yoon-Jung; Choi, Hye Jin; Kim, Sun Young (2018) Outpatient Palliative Care and Aggressiveness of End-of-Life Care in Patients with Metastatic Colorectal Cancer. The American journal of hospice & palliative care 35, 1, 166-172
165. Leeneman, B; Franken, M; Aarts, M J; van Akkooi, A C; van den Berkmortel, F W; van den Eertwegh, A J; de Groot, J W; Herbschleb, K H; van der Hoeven, K J; Hospers, G A; Kapiteijn, E; Piersma, D; van Rijn, R S; Suijkerbuijk, K P; ten Tije, A J; van der Veldt, A A; Vreugdenhil, G; Wouters, M W; van Zeijl, M C; Haanen, J B; Uyl-de Groot, C A (2018) End-of-Life Care in Patients with Metastatic Cutaneous Melanoma in the Netherlands. Value in Health 21, S51-S51
166. Lees, C.; Weerasinghe, S.; Lamond, N.; Younis, T.; Ramjeesingh, R. (2019) Palliative care consultation and aggressive care at end of life in unresectable pancreatic cancer. Current Oncology 26, 1, 28-36
167. Leon, X; Hitt, R; Constenla, M; Rocca, A; Stupp, R; Kovacs, A F; Amellal, N; Bessa, E H; Bourhis, J (2005) A retrospective analysis of the outcome of patients with recurrent and/or metastatic squamous cell carcinoma of the head and neck refractory to a platinum-based chemotherapy. Clinical oncology (Royal College of Radiologists (Great Britain)) 17, 6, 418-424
168. Lilley, Elizabeth J; Scott, John W; Goldberg, Joel E; Cauley, Christy E; Temel, Jennifer S; Epstein, Andrew S; Lipsitz, Stuart R; Smalls, Brittany L; Haider, Adil H; Bader, Angela M; Weissman, Joel S; Cooper, Zara (2018) Survival, Healthcare Utilization, and End-of-life Care Among Older Adults With Malignancy-associated Bowel Obstruction: Comparative Study of Surgery, Venting Gastrostomy, or Medical Management. Annals of Surgery 267, 4, 692-699
169. Lindemann, K. (2019) When to stop futile treatment towards end of life in gynaecological cancer patients: A population-based study in OSLO County, Norway. International Journal of Gynecological Cancer A18
170. Lissbrant, I F; Garmo, H; Widmark, A; Stattin, P (2013) Population-based study on use of chemotherapy in men with castration resistant prostate cancer. Acta Oncologica 52, 8, 1593-1601
171. Liu, Tsang-Wu; Chen, Jen-Shi; Wang, Hung-Ming; Wu, Shiao-Chi; Hung, Yen-Ni; Tang, Siew Tzuh (2009) Quality of end-of-life care between medical oncologists and other physician specialists for Taiwanese cancer decedents, 2001-2006. The oncologist 14, 12, 1232-1241
172. Llabres Valenti, E; Brenes, J; Hernandez, A; Ramchandani Vaswani, A; Beltra, L; Vicente, E; Cejuela, M; Rejas, D (2016) Antineoplastic therapy near the end of life: A retrospective analysis of the clinical practice in oncological adult patients. Annals of Oncology. Conference: 41st European Society for Medical Oncology Congress, ESMO 27
173. Lo Dico, S; Zecca, E; Brunelli, C; Bracchi, P; Vitali, M; Garassino, M; Caraceni, A (2017) Integration of palliative and oncology care in patients with lung and other thoracic cancer: Referral criteria and clinical care pathways. Annals of Oncology 28, vi89-vi90
174. Loggers, E; Fishman, P A; Ramaprasan, A; Earle, C (2012) Claims/utilization-based intensity of end-of-life (EOL) cancer care in integrated health systems (IHS). Journal of Clinical Oncology. Conference: ASCO's Quality Care Symposium 30, 34,
175. Lopes, F C; Guerra, N; Machado, M; Gloria, I; Brilhante, M; Carvalho, C; Braga, S (2013) Why do we treat lung cancer patients with chemotherapy until the end of life?. Journal of Thoracic Oncology 2, S1006-S1007
176. Lopez Acevedo, M; Havrilesky, L; Abernethy, A; Kamal, A; Broadwater, G; Berchuck, A; Lee, P (2013) Addressing comfort-care in the ambulatory setting is associated with im-proved end-of-life quality measures among women with ovarian cancer. Gynecologic Oncology 130, e29-e29
177. Low, D; Merkel, E C; Menon, M; Lyman, G H; Ddungu, H; Namukwaya, E; Leng, M; Casper, C (2017) Chemotherapy Use at the End of Life in Uganda. Journal of global oncology 3, 6, 711-719
178. Luthy, C; Pugliesi, A; Rapiti, E; Kossovsky, M; Dietrich, P Y; Cedraschi, C; Allaz, A F (2014) Aggressiveness of cancer treatment in patients hospitalized in a supportive care unit. Supportive Care in Cancer 23, 2, 325-331
179. Mack, J W; Chen, L H; Cooper, R M; Chao, C (2014) Intensity of end-of-life care among adolescents and young adults with cancer. Journal of Clinical Oncology. Conference 32, 15,
180. Mack, J W; Chen, K; Boscoe, F P; Gesten, F C; Roohan, P J; Schymura, M J; Schrag, D (2015) High intensity of end-of-life care among adolescent and young adult cancer patients in the New York State Medicaid program. Medical care 53, 12, 1018-1026
181. Mack, J W; Walling, A; Dy, S; Antonio, A L M; Adams, J; Keating, N L; Tisnado, D (2015) Patient beliefs that chemotherapy may be curative and care received at the end of life among patients with metastatic lung and colorectal cancer. Cancer 121, 11, 1891-1897
182. Magarotto, R; Lunardi, G; Coati, F; Cassandrini, P; Picece, V; Ferrighi, S; Oliosi, L; Venturini, M (2011) Reduced use of chemotherapy at the end of life in an integrated-care model of oncology and palliative care. Tumori 97, 5, 573-577
183. Mallett, V; Linehan, A; Burke, O; Laura, H; Sarah, P; Ryan, K; McCaffrey, J; Kelly, C; Higgins, M (2018) A review of cancer-directed treatments and palliative care provided to solid tumour oncology patients in the 12 weeks preceding death at an Irish University Hospital. Palliative Medicine 32, 10-10
184. Martins Branco, D; Lopes, S; Canario, R; Freire, J; Sousa, G; Lunet, N; Gomes, B (2017) Prevalence and recent time trend in aggressiveness of cancer care near the end of life: An expanded assessment in a cohort study. Annals of Oncology 28, v497-v497
185. Martoni, A A; Tanneberger, S; Mutri, V (2007) Cancer chemotherapy near the end of life: The time has come to set guidelines for its appropriate use. Tumori 93, 5, 417-422
186. Massa, I; Maltoni, M; Foca, F; Sansoni, E; Derni, S; Gentili, N; Valgiusti, M; Casadei Gardini, A; Pini, S; Amadori, D; Altini, M; Nanni, O (2015) Chemotherapy near end-of life: Aiming for appropriateness at the Cancer Institute of Romagna (IRST IRCCS). Annals of Oncology. Conference: 17th National Congress of Medical Oncology. Rome Italy. Conference Publication: 26
187. Massa, I; Nanni, O; Foca, F; Maltoni, M; Derni, S; Gentili, N; Frassineti, G L; Casadei Gardini, A; Valgiusti, M; Amadori, D; Prati, E; Altini, M; Gallegati, D; Sansoni, E (2018) Chemotherapy and palliative care near end-of life: Examining the appropriateness at a cancer institute for colorectal cancer patients. BMC Palliative Care 17, 86,
188. Massoudi, M; Balk, M; Yang, H; Bui, C N; Pandya, B J; Guo, J; Song, Y; Wu, E Q; Brown, B; Barlev, A; Flanders, S (2017) Number needed to treat and associated incremental costs of treatment with enzalutamide versus abiraterone acetate plus prednisone in chemotherapy-naive patients with metastatic castration-resistant prostate cancer. Journal of Medical Economics 20, 2, 121-128
189. Mathew, A; Achkar, T; Abberbock, S; Sandhu, G S; Jacob, M E; Villgran, V D; Rosenzweig, M Q; Puhalla, S; Brufsky, A M (2017) Prevalence and determinants of end-of-life chemotherapy use in patients with metastatic breast cancer. Breast Journal 23, 6, 718-722
190. Matter-Walstra, K; Achermann, R; Klingbiel, D; Bordoni, A; Dehler, S; Konzelmann, I; Jundt, G; Clough, K; Schwenkglenks, M; Pestalozzi, B (2013) Delivery of health care at the end of life in cancer patients from four Swiss cantons (SAKK 89/09). European Journal of Cancer 2, S325-S325
191. Matter-Walstra, K W; Achermann, R; Rapold, R; Klingbiel, D; Bordoni, A; Dehler, S; Jundt, G; Konzelmann, I; Clough-Gorr, K; Szucs, T; Pestalozzi, B C; Schwenkglenks, M (2014) Cancer-related therapies at the end of life in hospitalized cancer patients from four swiss cantons: SAKK 89/09. Oncology (Switzerland) 88, 1, 18-27
192. McCarthy, M (2015) Chemotherapy does not improve quality of life in cancer patients at end of life, US study finds. BMJ (Online) 351
193. McCracken, J A; Dabscheck, A; Coperchini, M; Hornung, I; Jalali, A; Akers, G; Karahalios, A; Gore, F; Lipton, L (2018) Prospective analysis of 30-day mortality following palliative chemotherapy at a tertiary cancer centre. Cancer Reports 1
194. McDermott, C L; Bansal, A; Ramsey, S D; Lyman, G H; Sullivan, S D (2018) Depression and Health Care Utilization at End of Life Among Older Adults With Advanced Non-Small-Cell Lung Cancer. Journal of Pain and Symptom Management 56, 5, 699-708.e1
195. McNaughton, C.; Lynch, S.; Newport, K. B.; Wong, R.; Svetec, S.; Moore, J.; Holliday, R.; Oyer, R. A.; Sivendran, S. (2018) Patterns of anti-cancer therapy use in the last 14 days of life in a community cancer institute. Journal of Clinical Oncology 36, 34,
196. McPherson, J. P.; Patel, S. B.; Igumnova, E.; Pettit, J.; Ose, D.; Haydell, T.; Martineau, J.; Meropol, N. J.; Beck, A. C. (2018) Real-time assessment of resource utilization and subsequent cost analysis in cancer patients (pts) near the end of life (EOL). Journal of Clinical Oncology 36, 30,
197. Mercado, F M R; Luhrs, C; Beal, A; Langdon, M; Secrest, J; Talbot, S M (2015) Integration of palliative care services into standard oncology practice at diagnosis of metastatic lung cancer at VA New York Harbor Healthcare System. Journal of Clinical Oncology. Conference 33, 29,
198. Merchant, S J; Brogly, S B; Goldie, C; Booth, C M; Nanji, S; Patel, S V; Lajkosz, K; Baxter, N N (2018) Palliative Care is Associated with Reduced Aggressive End-of-Life Care in Patients with Gastrointestinal Cancer. Annals of Surgical Oncology 25, 6, 1478-1487
199. Merlo, Domenico Franco; Beccaro, Monica; Costantini, Massimo; Italian Survey of the Dying of Cancer Study, Group (2008) An unconventional cancer treatment lacking clinical efficacy remains available to Italian cancer patients. Tumori 94, 6, 830-832
200. Merth, G; A, R; Mokrai, D; Ruzsa, V; Fuleki, G; Halmai, L; Rozsa, P (2018) Palliative Treatment and Its Cost in the Last Six Months of Life for Metastatic Colorectal Cancer Patients. Value in Health 21, S31-S31
201. Michael, N.; Beale, G.; O'Callaghan, C.; Melia, A.; Desilva, W.; Costa, D.; Kissane, D.; Shapiro, J.; Hiscock, R. (2019) Timing of palliative care referral and aggressive cancer care toward the end-of-life in pancreatic cancer: A retrospective, single-center observational study. BMC Palliative Care 18, 1,
202. Mieras, A. (2020) Chemotherapy and Tyrosine Kinase Inhibitors in the last month of life in patients with metastatic lung cancer: A patient file study in the Netherlands. Eur J Cancer Care (Engl) e13210
203. Miesfeldt, Susan; Murray, Kimberly; Lucas, Lee; Chang, Chiang-Hua; Goodman, David; Morden, Nancy E (2012) Association of age, gender, and race with intensity of end-of-life care for Medicare beneficiaries with cancer. Journal of Palliative Medicine 15, 5, 548-554
204. Mohammed, A A; Al-Zahrani, A S; Ghanem, H M; Farooq, M U; El Saify, A M; El-Khatib, H M (2015) End-of-life palliative chemotherapy: Where do we stand?. Journal of the Egyptian National Cancer Institute 27, 1, 35-39
205. Moltara, M E; Mesti, T; Mrsnik, M; Ivanetic, M; Cervek, M; Rajer, M; Zavratnik, B; Unk, M; Blas, M; Tonkli, A; Ravnik, M; Horvat, M; Gregoric, B; Pelipenko, K; Zakotnik, B; Cervek, J (2012) Anticancer treatment near the end of life. Supportive Care in Cancer 1, S166-S166
206. Monier, P A; Chrusciel, J; Sanchez, S; Laval, G; Barbaret, C (2018) Association between palliative care follow-up and aggressiveness of cancer care near the end of life. Palliative Medicine 32, 11-11
207. Morin, L; Aubry, R; Beaussant, Y; Rochigneux, P; Goldwasser, F; Tournigand, C (2015) Burden of inpatient care and treatments in terminallyill cancer patients: Results from a population-based, retrospective study from administrative data in France. Journal of Clinical Oncology. Conference 33, 15,
208. Morin, L; Beaussant, Y; Aubry, R; Fastbom, J; Johnell, K (2016) Aggressiveness of end-of-life care for hospitalized cancer patients with and without dementia: A nationwide matched cohort study in france. Palliative Medicine 30, NP167-NP167
209. Morishima, T; Lee, J; Otsubo, T; Imanaka, Y (2014) Association of healthcare expenditures with aggressive versus palliative care for cancer patients at the end of life: A cross-sectional study using claims data in japan. International Journal for Quality in Health Care 26, 1, 79-86
210. Mrad, C; Abougergi, M S; Daly, R M (2017) Trends in aggressive care at the end-of-life for stage IV lung cancer patients. Journal of Clinical Oncology. Conference 35, 15,
211. Muetherig, A; Sockel, K; Rentsch, A; Folprecht, G (2015) Mortality from outpatients chemotherapy (CTx) in patients (pts) with solid tumors. Journal of Clinical Oncology. Conference 33, 15,
212. Murillo Jr., Jose R; Koeller, Jim (2006) Chemotherapy given near the end of life by community oncologists for advanced non-small cell lung cancer. The oncologist 11, 10, 1095-1099
213. Namireddy, P; Macherla, S; McClain, J T; Muzafiar, M (2017) Racial and social disparities on aggressiveness of end-of-life cancer care in a rural academic center. Journal of Clinical Oncology. Conference 35, 15,
214. Nappa, U; Axelsson, B (2010) Proportion of incurable cancer patients who receive palliative chemotherapy during the last month of life. Palliative Medicine 1, S78-S78
215. Nappa, U; Lindqvist, O; Rasmussen, B H; Axelsson, B (2016) Routine assessment of performance status during palliative chemotherapy when approaching end-of-life. European journal of oncology nursing : the official journal of European Oncology Nursing Society 21, 266-271
216. Neubauer, M A; Garey, J S; Turnwald, B; Harrell, R K; Howell, J; Hayes, J; Bhowmik, D; Russell Hoverman, J J; Don Brooks, B; Beveridge, R (2013) Impact of pathways on chemotherapy administration before death. Journal of Clinical Oncology. Conference 31, 15,
217. Nevadunsky, N S; Rivera, E; Eti, S; Rapkin, B; Selwyn, P; Goldberg, G L (2012) End-of-life care in a racially and ethnically diverse population of women with gynecologic malignancies-a pilot study. Supportive Care in Cancer 1, S189-S190
218. Nevadunsky, N; Gordon, S; Spoozak, L; Harris, K; Rivera, E; Van Arsdale, A; Rapkin, B; Selwyn, P; Goldberg, G (2013) Results of timely palliative medicine consultation on end-of-life care outcomes for women with gynecologic malignancies. Gynecologic Oncology 130, e8-e8
219. Newport, K; Holliday, R; McNaughton, C; Gehron, E; Sivendran, M H S (2017) A description of cancer directed therapy within 14 days of death in a community cancer institute. Journal of Clinical Oncology 35, 73-73
220. Nguyen, M; Shum, E; Ng Ying Kin, S; Wann, A; Tamjid, B; Torres, J (2018) Anticancer therapy within the last 30 days of life in a regional cancer centre. Supportive Care in Cancer 26, S385-S385
221. Nguyen, M. (2020) Anticancer therapy within the last 30 days of life: results of an audit and re-audit cycle from an Australian regional cancer centre. BMC Palliat Care 14
222. Nieder, C; Tollali, T; Norum, J; Pawinski, A; Bremnes, R M (2012) A population-based study of the pattern of terminal care and hospital death in patients with non-small cell lung cancer. Anticancer Research 32, 1, 189-194
223. Nieder, C; Tollali, T; Dalhaug, A; Haukland, E; Aandahl, G; Pawinski, A; Norum, J (2014) Active anticancer treatment during the final month of life in patients with non-small cell lung cancer. Anticancer Research 34, 2, 1015-1020
224. Nieder, C; Tollali, T; Haukland, E; Reigstad, A; Flatoy, L R; Engljahringer, K (2016) Impact of early palliative interventions on the outcomes of care for patients with non-small cell lung cancer. Supportive Care in Cancer 24, 10, 4385-4391
225. Nieder, C; Haukland, E; Mannsaker, B; Pawinski, A; Yobuta, R; Norum, J (2019) Initiation of Systemic Therapy During the Last 30 Days of Life in Patients With Metastatic Castration-resistant Prostate Cancer. Anticancer Research 39, 1, 335-340
226. Nipp, R D; Tramontano, A C; Kong, C Y; Hur, C (2018) Patterns and predictors of end-of-life care in older patients with pancreatic cancer. Cancer Medicine 7, 12, 6401-6410
227. Nitecki, R.; Bercow, A.; Gockley, A. A.; Growdon, W. B. (2019) Clinical trial participation and measures of aggressive care at the end of life in patients with ovarian cancer. Gynecologic Oncology 153, 3, e10
228. Nitecki, R. (2020) Clinical trial participation and aggressive care at the end of life in patients with ovarian cancer. Int J Gynecol Cancer 201-206
229. Numico, G; Trogu, A; Cristofano, A; Mozzicafreddo, A; Courthod, G; Franco, P; Silvestris, N (2014) Active treatment given in the last weeks of life: poor quality cancer care or justifiable behavior?. Supportive Care in Cancer 22, 10, 2813-2819
230. Ortiz, J S (2012) Chemotherapy at the end of life: Up until when?. Clinical and Translational Oncology 14, 9, 667-674
231. Pacetti, P; Paganini, G; Orlandi, M; Mambrini, A; Pennucci, M C; Del Freo, A; Cantore, M (2015) Chemotherapy in the last 30 days of life of advanced cancer patients. Supportive Care in Cancer 23, 11, 3277-3280
232. Paiva, C E; De Oliveira Valentino, T C; Sakamoto, B; Paiva, R (2016) Impact of palliative care (PC) on aggressive end-of-life (EOL) care indicators among advanced cancer patients (ACPs). Journal of Clinical Oncology. Conference 34
233. Palaia, I. (2019) The EOLO (End-of-Life Ovarian Cancer) Study: Approach to Ovarian Cancer Patients at the End of Life. Oncology 306-310
234. Palmieri, L. (2019) Reasons for chemotherapy discontinuation and end of life in gastrointestinal cancers: A multicentric prospective AGEO study. Annals of Oncology aa99-aa100
235. Paque, K; Elseviers, M; Vander Stichele, R; Pardon, K; Hjermstad, M J; Kaasa, S; Dilles, T; De Laat, M; Van Belle, S; Christiaens, T; Deliens, L (2018) Changes in medication use in a cohort of patients with advanced cancer: The international multicentre prospective European Palliative Care Cancer Symptom study. Palliative Medicine 32, 4, 775-785
236. Parekh, H D; Tullio, K; Elson, P; Davis, M P; Velcheti, V; Stevenson, J; Shapiro, M A; Carrino, C M; Pennell, N A (2016) The effect of routine early palliative care (PC) consultation on aggressiveness of care at the end of life (EOL) in patients with advanced non-small cell lung cancer (NSCLC). Journal of Clinical Oncology. Conference 34
237. Parikh, A R; Kim, B; Pantoja, P; Tisnado, D M; Ahluwalia, S C; Walling, A M; Asch, S M; Lorenz, K (2013) How is KRAS testing associated with treatment and supportive care for patients with metastatic colorectal cancer? VA national assist project. Journal of Clinical Oncology. Conference: ASCO's Quality Care Symposium 31, 31,
238. Parikh, R B; Elffky, A; Pany, M J; Obermeyer, Z (2017) A machine learning approach to predicting short-term mortality risk for patients starting chemotherapy. Journal of Clinical Oncology. Conference 35, 15,
239. Park, M; Song, I (2018) Medical care costs of cancer in the last year of life using national health insurance data in Korea. PLoS ONE 13
240. Pataky, R E; Cheung, W Y; de Oliveira, C; Bremner, K E; Chan, K K W; Hoch, J S; Krahn, M D; Peacock, S J (2016) Population-based trends in systemic therapy use and cost for cancer patients in the last year of life. Current Oncology 23, S32-S41
241. Paul, S; Sanders, S A; Stenehjem, D D (2014) Utilization of antineoplastic chemotherapy near the end of life in patients with solid tumors. Journal of Clinical Oncology. Conference 32, 15,
242. Perng, P; Saleemi, S; Alcorn, S R; Ellsworth, S G; McNutt, T R; Hales, R K; DeWeese, T L; Smith, T J (2014) Patterns of chemotherapy near the end of life for patients receiving palliative bone radiotherapy. Journal of Clinical Oncology. Conference 32, 15,
243. Petrillo, L. A. (2020) Performance status and end-of-life care among adults with non-small cell lung cancer receiving immune checkpoint inhibitors. Cancer 2288-2295
244. Phelps, A; Nilsson, M; Balboni, T; Wright, A; Paulk, E; Trice, E; Peteet, J; Block, S; Schrag, D; Prigerson, H (2009) Religious coping among advanced cancer patients and its associations with end-of-life treatment preferences and receipt of intensive life-prolonging care. Psycho-Oncology 1, S27-S28
245. Phelps, A C; Zhang, B; Prigerson, H G (2012) Clinical trial participation as part of end-of-life (EOL) cancer care: Associations with medical care near death and bereaved caregivers' mental health. Journal of Clinical Oncology. Conference 30, 15,
246. Philip, J; Hudson, P; Bostanci, A; Street, A; Horey, D E; Aranda, S; Zordan, R; Rumbold, B D; Moore, G; Sundararajan, V (2015) Metastatic non-small cell lung cancer: A benchmark for quality end-of-life cancer care?. Medical Journal of Australia 202, 3, 139-144
247. Philip, J; Collins, A; Burchell, J L; Mileshkin, L; Le, B; Hudson, P; McLachlan, S A; Currow, D; Millar, J; Krishnasamy, M; Sundararajan, V (2016) The quality of end of life care of patients with metastatic small cell lung cancer: Does it differ from other lung cancer patients?. Palliative Medicine 30, 6, NP55-NP56
248. Pirl, W F; Greer, J A; Irwin, K; Lennes, I T; Jackson, V A; Park, E R; Fujisawa, D; Wright, A A; Temel, J S (2014) Processes of discontinuing chemotherapy for metastatic non-small cell lung cancer at the end of life. Journal of Clinical Oncology. Conference: Palliative Care in Oncology Symposium 32, 31,
249. Pitson, G.; Matheson, L.; Garrard, B.; Eastman, P.; Rogers, M. (2020) Population-Based Analysis of Radiotherapy and Chemotherapy Treatment in the Last Month of Life within regional Australia. Internal medicine journal
250. Presley, C. J. (2020) Concurrent Hospice Care and Cancer-Directed Treatment for Advanced Lung Cancer and Receipt of Aggressive Care at the End of Life in the Veteran's Health Administration. J Palliat Med 1038-1044
251. Prigerson, H G; Bao, Y; Shah, M A; Paulk, M E; LeBlanc, T W; Schneider, B J; Garrido, M M; Reid, M C; Berlin, D A; Adelson, K B; Neugut, A I; Maciejewski, P K (2015) Chemotherapy Use, Performance Status, and Quality of Life at the End of Life. JAMA oncology 1, 6, 778-784
252. Prommer, E; Dy, S; Billing, L; Buss, M; Smith, T (2012) Chemotherapy in the last two weeks of life: When is it appropriate? When is it not appropriate? Cancer SIG. Journal of Pain and Symptom Management 43, 2, 365-366
253. Qiu, M Z; Xu, F; Wang, S S; Luo, H Y; Wang, F; Li, F H; Sun, X F; Xu, G C; Lin, T Y; Huang, H Q; Jiang, W Q; Guan, Z Z; Xu, R H (2007) Responses of 109 adult soft tissue sarcoma patients to chemotherapy. [Chinese]. Ai zheng = Aizheng = Chinese journal of cancer 26, 12, 1344-1349
254. Randen, M; Helde-Frankling, M; Runesdotter, S; Strang, P (2013) Treatment decisions and discontinuation of palliative chemotherapy near the end-of-life, in relation to socioeconomic variables. Acta Oncologica 52, 6, 1062-1066
255. Rautakorpi, L K; Seyednasrollah, F; Makela, J M; Hirvonen, O M; Laitinen, T; Elo, L L; Jyrkkio, S M (2017) End-of-life chemotherapy use at a Finnish university hospital: a retrospective cohort study. Acta Oncologica 56, 10, 1272-1276
256. Rintanen, V; Anttila, M; Selander, T; Sallinen, H (2016) Aggressive care at the end of life has no impact on survival in patients with ovarian cancer. International Journal of Gynecological Cancer 26, 950-950
257. Rochigneux, P; Raoul, J L; Beaussant, Y; Aubry, R; Goldwasser, F; Tournigand, C; Morin, L (2017) Use of chemotherapy near the end of life: What factors matter?. Annals of Oncology 28, 4, 809-817
258. Rodriguez, M A; Cheng, L; De Jesus, A Y; Ferrajoli, A; Burke, T W (2012) Chemotherapy use within the last 14 days of life in patients with hematological malignancies. Blood. Conference: 54th Annual Meeting of the American Society of Hematology, ASH 120, 21,
259. Rodriguez, M A; Cheng, L; DeJesus, A Y; Burke, T W (2012) Chemotherapy within last 30 days of life: Differences between hematologic malignancy (HM) and solid tumor (ST) patients at a cancer hospital. Cancer Research. Conference: 103rd Annual Meeting of the American Association for Cancer Research, AACR 72, 8,
260. Rodriguez, M A; DeJesus, Y; Cheng, L; Burke, T W (2012) Chemotherapy within the last 30 days of life among patients with metastatic solid tumors. Journal of Clinical Oncology. Conference 30, 15,
261. Rodriguez, M A; DeJesus, Y A; Cheng, L; Buzdar, A; Burke, T W (2013) Factors related to end-of-life (EOL) chemotherapy in solid tumor (ST) patients. Journal of Clinical Oncology. Conference: ASCO's Quality Care Symposium 31, 31,
262. Romano, A M; Gade, K E; Nielsen, G; Havard, R; Harrison, J H; Barclay, J; Stukenborg, G J; Read, P W; Blackhall, L J; Dillon, P M (2017) Early palliative care reduces end-of-life intensive care unit (ICU) use but not ICU course in patients with advanced cancer. Oncologist 22, 3, 318-323
263. Roncolato, F T; Joly, F; O'Connell, R; Lanceley, A; Hilpert, F; Buizen, L; Okamoto, A; Aotani, E; Pignata, S; Donnellan, P; Oza, A; Avall-Lundqvist, E; Berek, J S; Heitz, F; Feeney, A; Berton-Rigaud, D; Stockler, M R; King, M; Friedlander, M (2017) Reducing uncertainty: Predictors of stopping chemotherapy early and shortened survival time in platinum resistant/refractory ovarian cancer-The GCIG symptombenefit study. Oncologist 22, 9, 1117-1124
264. Rozman, L M; Campolina, A G; Lopez, R V M; Kobayashi, S T; Chiba, T; De Soarez, P C (2018) Early Palliative Care and Its Impact on End-of-Life Care for Cancer Patients in Brazil. Journal of Palliative Medicine 21, 5, 659-664
265. Saito, A M; Landrum, M; Neville, B A; Ayanian, J Z; Earle, C C (2011) The effect on survival of continuing chemotherapy to near death. BMC Palliative Care 10, 14,
266. Samuelson, C V; Griffin, M; Welch, E; Went, R; Kaul, S; Ng, J P; Barker, H; Snowden, J A (2016) Mortality within 30 days of systemic anticancer therapy (SACT)-results of a multi-site audit following the NCEPOD model in the South Yorkshire region. British Journal of Haematology 173, 34-34
267. Sánchez-Cuervo, M. (2020) Chemotherapy Near the End of Life in Onco-Hematological Adult Patients. Am J Hosp Palliat Care 641-647
268. Sano, M; Fushimi, K (2017) Association of Palliative Care Consultation With Reducing Inpatient Chemotherapy Use in Elderly Patients With Cancer in Japan: Analysis Using a Nationwide Administrative Database. The American journal of hospice & palliative care 34, 7, 685-691
269. Santana-Davila, R; Kelley, M J; Williams, C D; Eaton, K; Whittle, J C (2015) Chemotherapy at the end of life (EOL) for patients with lung cancer within the VA system. Journal of Thoracic Oncology 2, S789-S790
270. Santos Pérez, M. I. (2019) [Chemotherapy at the end of life: Uncommon clinical practice?]. J Healthc Qual Res 201-207
271. Sato, K; Miyashita, M; Morita, T; Sanjo, M; Shima, Y; Uchitomi, Y (2008) Quality of end-of-life treatment for cancer patients in general wards and the palliative care unit at a regional cancer center in Japan: A retrospective chart review. Supportive Care in Cancer 16, 2, 113-122
272. Sato, Y; Miyashita, M; Sato, K; Fujimori, K; Ishikawa, K B; Horiguchi, H; Fushimi, K; Ishioka, C (2018) End-of-life care for cancer patients in Japanese acute care hospitals: A nationwide retrospective administrative database survey. Japanese journal of clinical oncology 48, 10, 877-883
273. Schulkes, K J G; van Walree, I C; van Elden, L J R; van den Bos, F; van Huis-Tanja, L; Lammers, J W J; Ten Bokkel Huinink, D; Hamaker, M E (2018) Chemotherapy and healthcare utilisation near the end of life in patients with cancer. European journal of cancer care 27, 2, e12796-e12796
274. Sedhom, R; Sedhom, D; Barile, D (2017) A comparison of quality end-of-life care in patients with hematologic and solid malignancies: Identifying deficiencies in patient care. Journal of General Internal Medicine 32, S92-S92
275. Sezgin Goksu, Sema; Gunduz, Seyda; Unal, Dilek; Uysal, Mukremin; Arslan, Deniz; Tatli, Ali M; Bozcuk, Hakan; Ozdogan, Mustafa; Coskun, Hasan S (2014) Use of chemotherapy at the end of life in Turkey. BMC Palliative Care 13, 1, 51-51
276. Sharma, G; Wang, Y; Graham, J E; Kuo, Y F; Goodwin, J S (2013) Provider Continuity Prior to the Diagnosis of Advanced Lung Cancer and End-of-Life Care. PLoS ONE 8
277. Sheffield, Kristin M; Boyd, Casey A; Benarroch-Gampel, Jamie; Kuo, Yong-Fang; Cooksley, Catherine D; Riall, Taylor S (2011) End-of-life care in Medicare beneficiaries dying with pancreatic cancer. Cancer 117, 21, 5003-5012
278. Sheng, Jin; Zhang, Ya-Xiong; He, Xiao-Bo; Fang, Wen-Feng; Yang, Yun-Peng; Lin, Gui-Nan; Wu, Xuan; Li, Ning; Zhang, Jing; Zhai, Lin-Zhu; Zhao, Yuan-Yuan; Huang, Yan; Zhou, Ning-Ning; Zhao, Hong-Yun; Zhang, Li (2017) Chemotherapy Near the End of Life for Chinese Patients with Solid Malignancies. The oncologist 22, 1, 53-60
279. Shim, H J; Yun, J Y; Hwang, J E; Bae, W K; Cho, S H; Chung, I J (2011) Prognostic factor analysis of third-line chemotherapy in patients with advanced gastric cancer. Gastric Cancer 14, 3, 249-256
280. Silverman, R; Smith, L; Sundar, S (2014) Benchmarking 30 Day Mortality After Palliative Chemotherapy for Solid Tumours. Clinical Oncology 26, 4, 236-240
281. Sivendran, S.; Lynch, S.; McNaughton, C.; Wong, R.; Svetec, S.; Moore, J. H.; Holliday, R.; Oyer, R. A.; Newport, K. (2019) Anticancer Therapy at the End of Life: Lessons From a Community Cancer Institute. Journal of palliative care 825859719851484
282. Skov Benthien, K; Adsersen, M; Petersen, M A; Soelberg Vadstrup, E; Sjogren, P; Groenvold, M (2018) Is specialized palliative cancer care associated with use of antineoplastic treatment at the end of life? A population-based cohort study. Palliative Medicine 32, 9, 1509-1517
283. Smith, C E P; Coke, P; Kluger, M; Kamal, A; Kelley, M J (2018) National trends in end of life care for veterans with advanced cancer. Journal of Clinical Oncology. Conference 36, 30,
284. Soares, L. G. L. (2019) Trends in Health-Care Utilization at the End of Life Among Patients With Hematologic Malignancies in a Middle-Income Country: Challenges and Opportunities in Brazil. Am J Hosp Palliat Care 775-779
285. Soares, L. G. L. (2020) Quality Indicators of End-of-Life Care Among Privately Insured People With Cancer in Brazil. Am J Hosp Palliat Care 594-599
286. Soh, T I P; Yuen, Y C; Teo, C; Lim, S W; Chan, N; Wong, A S C (2012) Targeted therapy at the end of life in advanced cancer patients. Journal of Palliative Medicine 15, 9, 991-997
287. Stavas, M J; Martin, S F; Phillips, S E; Perkins, S M; Shinohara, E T (2015) The utilization of chemotherapy and radiation at the end of life in individuals with metastatic non-small cell lung cancer. Journal of Clinical Oncology. Conference 33, 29,
288. Strasser, F; Blum, D; Widmer, C; Silzle, T; De Wolf-Linder, S; Nestor, K; Omlin, A; Schmitz, N; Scherrer, A; Fruh, M; Koberle, D (2011) Development of service models of integrated oncology and palliative care: Temporal aspects, oncologists' role and chemotherapy use. Onkologie 6, 28-28
289. Stuver, S O; McNiff, K; Fraile, B; Odejide, O; Abel, G A; Dodek, A; Jacobson, J O (2016) Novel Data Sharing Between a Comprehensive Cancer Center and a Private Payer to Better Understand Care at the End of Life. Journal of Pain and Symptom Management 52, 2, 161-169
290. Taberner Bonastre, P; Taberner Bonastre, M T; Soler Company, E; Perez-Serrano Lainosa, M D (2016) Chemotherapy near the end of life; assessment of the clinical practise in onco-hematological in adult patients. Farmacia hospitalaria : organo oficial de expresion cientifica de la Sociedad Espanola de Farmacia Hospitalaria 40, 1, 14-24
291. Tancredi, R; Stefani, S; Gervaso, L; Fregoni, V; Poojary, P; Cefali, M; Piacentini, G; Riccardi, A (2017) End-of-life chemotherapy and adherence to ASCO's Top Five List recommendations: A single-center retrospective review. Journal of Clinical Oncology. Conference 35, 15,
292. Tang, S; Liu, T W; Chen, J S; Wang, H M; Wu, S C; Hung, Y N (2009) Performance of medical oncologists on end-of-life care for Taiwanese cancer decedents, 2001-2006. European Journal of Cancer, Supplement 7, 179-179
293. Tang, S T; Wu, S C; Hung, Y N; Huang, E W; Chen, J S; Liu, T W (2009) Trends in quality of end-of-life care for Taiwanese cancer patients who died in 2000-2006. Annals of Oncology 20, 2, 343-348
294. Tang, S T; Liu, T W; Chang, W C; Wang, H M; Chen, J S; Koong, S L; Hsiao, S C (2011) Use of chemotherapy at the end of life among taiwanese cancer decedents, 2001-2006. European Journal of Cancer 1, S229-S230
295. Tang, S T; Liu, T W; Shyu, Y I L; Huang, E W; Koong, S L; Hsiao, S C (2012) Impact of age on end-of-life care for adult Taiwanese cancer decedents, 2001-2006. Palliative Medicine 26, 1, 80-88
296. Tanguy-Melac, A. (2019) Health care utilization by men with prostate cancer during the year before their death: A 2015 population-based study. Prog Urol 995-1006
297. Tanguy-Melac, A. (2020) Intensity of care, expenditure, place and cause of death people with lung cancer in the year before their death: A French population based study. Bull Cancer 308-321
298. Tassinari, D; Panzini, I; Fabbri, E; Cherubini, C; Gallegati, D; Rossi, L; Nanni, O; Massa, I; Maltoni, M (2016) Trend in treatment aggressiveness at the end of life in an Italian homogeneous area: Preliminary data. Journal of Clinical Oncology. Conference 34
299. Temel, J S; McCannon, J; Greer, J A; Jackson, V A; Ostler, P; Pirl, W F; Lynch, T J; Billings, J A (2008) Aggressiveness of care in a prospective cohort of patients with advanced NSCLC. Cancer 113, 4, 826-833
300. Thomas, S P; Rice, M A; T.-T, A Ho; Heard, B; Harper, J; Fishkin, P A S (2013) Evaluation of chemotherapy within last two weeks of life: Patterns of care. Journal of Clinical Oncology. Conference 31, 15,
301. Thompson, C A; Hugo, S E; Swetz, K M; Novotny, P J; Sloan, J A; Loprinzi, C L; Moynihan, T J; Shanafelt, T D (2013) End-of-life care in a population-based cohort of cancer patients: clinical trial participation versus standard of care. BMJ supportive & palliative care 3, 2, 181-187
302. Tramontano, A. C. (2018) Hospice use and end-of-life care among older patients with esophageal cancer. Health Science Reports
303. Triplett, D P; LeBrett, W G; Bryant, A K; Bruggeman, A R; Matsuno, R K; Hwang, L; Boero, I J; Roeland, E J; Yeung, H N; Murphy, J D (2017) Effect of palliative care on aggressiveness of end-of-life care among patients with advanced cancer. Journal of Oncology Practice 13, 9, e760-e769
304. Tsai, Hsin-Yun; Chung, Kuo-Piao; Kuo, Raymond Nien-Chen (2018) Impact of Targeted Therapy on the Quality of End-of-Life Care for Patients With Non-Small-Cell Lung Cancer: A Population-Based Study in Taiwan. Journal of Pain and Symptom Management 55, 3, 798-807.e4
305. Tsumura, A; Noguchi-Abe, K; Ishida, Y (2018) Quality of end of life care in adolescent and young adults with cancer in Japan. Supportive Care in Cancer 26, S386-S386
306. Turker, I; Komurcu, S; Arican, A; Doruk, H; Ozyilkan, O; Coskun, H S; Colak, D s; Ucgul Cavusoglu, E; Ata, A; Sezer, A; Yesil Cinkir, H; Senler, F C; Arpaci, F (2014) Investigational tests and treatments performed in terminal stage cancer patients in two weeks before death: Turkish oncology group (TOG) study. Medical Oncology 31, 12, 1-6
307. Urvay, S. (2020) Chemotherapy at the End of Life. J Palliat Care 825859720946505
308. van Herk-Sukel, Myrthe P P; van de Poll-Franse, Lonneke V; Creemers, Geert-Jan; Lemmens, Valery E P P; van der Linden, Paul D; Herings, Ron M C; Coebergh, Jan Willem W; Voogd, Adri C (2013) Major changes in chemotherapy regimens administered to breast cancer patients during 2000-2008 in the Netherlands. The breast journal 19, 4, 394-401
309. Verma, V.; Brian Butler, E.; Teh, B. S.; Haque, W. (2019) Patterns of End-of-Life Oncologic Care for Stage IV Non-small Cell Lung Cancer in the United States. Anticancer Research 39, 6, 3137-3140
310. Viel, E; Chaigneau, L; Fanton, E; Kalbacher, E; Thiery-Vuillemnin, A; Villanueva, C; Dobi, E; Curtit, E; Almotlak, H; Aubry, R; Pivot, X (2013) Specific anticancer treatments in the last 3 months of life: A French experience. Supportive Care in Cancer 21, 2, 405-412
311. Von Gruenigen, V; Daly, B; Gibbons, H; Hutchins, J; Green, A (2008) Indicators of survival duration in ovarian cancer and implications for aggressiveness of care. Cancer 112, 10, 2221-2227
312. Walter, J; Tufman, A; Holle, R; Schwarzkopf, L (2017) Comparison of costs and care of lung cancer patient at the end-of-life in germany depending on the time of survival after diagnosis. Value in Health 20, A511-A511
313. Walter, J; Tufman, A; Leidl, R; Holle, R; Schwarzkopf, L (2018) Rural versus urban differences in end-of-life care for lung cancer patients in Germany. Supportive Care in Cancer 26, 7, 2275-2283
314. Wang, S Y; Hall, J; Long, J B; Pollack, C E; Bradley, E; Adelson, K B; Gross, C P (2015) Trends and regional variation of end-of-life cancer care in the Medicare program. Journal of Clinical Oncology. Conference 33, 15,
315. Wang, Shi-Yi; Hall, Jane; Pollack, Craig E; Adelson, Kerin; Bradley, Elizabeth H; Long, Jessica B; Gross, Cary P (2016) Trends in end-of-life cancer care in the Medicare program. Journal of Geriatric Oncology 7, 2, 116-125
316. Warren, J L; Barbera, L; Bremner, K E; Yabroff, K R; Hoch, J S; Barrett, M J; Luo, J; Krahn, M D (2011) End-of-life care for lung cancer patients in the United States and Ontario. Journal of the National Cancer Institute 103, 11, 853-862
317. Watanabe, K; Nagamata, M; Yomota, M; Zenke, Y; Okuma, Y; Hosomi, Y; Okamura, T (2017) Administration of chemotherapy to advanced lung cancer patients near the end of life. Annals of Oncology 28, ix83-ix83
318. Webster, J; Scott, J; Andrew Johnson, K (2018) Impact of chemotherapy provided to patients late in life on resource utilization and cost of care. Journal of Clinical Oncology. Conference 36, 15,
319. Wheatley-Price, P; Ali, M; Balchin, K; Spencer, J; Fitzgibbon, E; Cripps, C (2014) The role of palliative chemotherapy in hospitalized patients. Current Oncology 21, 4, 187-192
320. Wong, L C; Khattak, M N (2010) Literature search on end-of-life aggressiveness of care studies with respect to anticancer treatments. Annals of Oncology 8, viii366-viii366
321. Wong, A S; Teo, C; Lim, S W; Wong, E; Soo, R A; Chan, N (2010) Targeted therapy at the end of life for patients with lung cancer. Journal of Palliative Medicine 13, 8, 945-948
322. Wright, A A; Prigerson, H G (2011) Palliative chemotherapy: Does aggressive care beget aggressive care?. Journal of Clinical Oncology. Conference: ASCO Annual Meeting 29, 15,
323. Wright, A A; Earle, C; Keating, N L (2013) End-of-life care for Medicare beneficiaries with ovarian cancer: Evaluation of intensity and rate of hospitalizations. Journal of Clinical Oncology. Conference 31, 15,
324. Wright, A A; Zhang, B; Keating, N L; Weeks, J C; Prigerson, H G (2014) Associations between palliative chemotherapy and adult cancer patients' end of life care and place of death: Prospective cohort study. BMJ (Online) 348
325. Wu, E; Rogers, A; Ji, L; Sposto, R; Mittra, A; Church, T; Tripathy, D; Lin, Y G (2012) End-of-life health care utilization of gynecologic cancer patients at an urban, public hospital. Gynecologic Oncology 127, S12-S12
326. Wu, Chin-Chia; Hsu, Ta-Wen; Chang, Chun-Ming; Lee, Cheng-Hung; Huang, Chih-Yuan; Lee, Ching-Chih (2016) Palliative Chemotherapy Affects Aggressiveness of End-of-Life Care. The oncologist 21, 6, 771-777
327. Yabroff, K; Warren, J; Barbera, L C; Bremner, K; Hoch, J; Barrett, M; Luo, J; Krahn, M (2010) End-of-life care for elderly patients with advanced lung cancer in the United States and Ontario. Journal of Clinical Oncology. Conference 28, 15,
328. Yang, D; Qiu, M; Zou, L Q; Zhang, W; Jiang, Y; Zhang, D Y; Yan, X (2013) The role of palliative chemotherapy for terminally ill patients with advanced NSCLC. Thoracic Cancer 4, 2, 153-160
329. Yang, A. (2020) Racial Disparities in Health Care Utilization at the End of Life Among New Jersey Medicaid Beneficiaries With Advanced Cancer. JCO Oncol Pract e538-e548
330. Yoo, S H; Keam, B; Kim, M; Kim, T M; Kim, D W; Heo, D S (2018) The effect of hospice consultation on aggressive treatment of lung cancer. Cancer Research and Treatment 50, 3, 720-728
331. Yun, Young Ho; Kwak, Miyoung; Park, Sang Min; Kim, Samyong; Choi, Jong Soo; Lim, Ho-Yeong; Lee, Chang Geol; Choi, Youn Seon; Hong, Young Seon; Kim, Si-Young; Heo, Dae Seog (2007) Chemotherapy use and associated factors among cancer patients near the end of life. Oncology 72, 3, 164-171
332. Zachariah, F.; Morse, D.; Kinsey, L.; Loscalzo, M.; Koczywas, M.; Dale, W. (2019) The Integrated Care Service: Impact of a Multidisciplinary Supportive Care Service on Hospice Discharge Rates for Medical Oncology Patients in a NCI-Designated Cancer Center (SA511B). Journal of Pain and Symptom Management 57, 2, 448
333. Zakaria, A S; Santos, F; Dragomir, A; Kassouf, W; Tanguay, S; Aprikian, A (2017) Health care services utilization during the last 6 months of life among patients with bladder cancer who underwent radical cystectomy in Quebec, Canada. Urologic Oncology: Seminars and Original Investigations 35, 9, 539.e1-539.e7
334. Zakhour, M; Labrant, L; Rimel, B J; Walsh, C; Li, A J; Karlan, B Y; Cass, I (2014) Too much, too late: Aggressive measures and the timing of end-of life care discussions in women with gynecologic malignancies. Gynecologic Oncology 1, 54-54
335. Zdenkowski, N; Cavenagh, J; Ku, Y C; Bisquera, A; Bonaventura, A (2013) Administration of chemotherapy with palliative intent in the last 30 days of life: The balance between palliation and chemotherapy. Internal Medicine Journal 43, 11, 1191-1198
336. Zerillo, J A; Smith, A K; Schonberg, M A; McCarthy, E P (2011) The influence of age on intensity of care at the end of life among older patients with advanced cancer. Journal of Clinical Oncology. Conference: ASCO Annual Meeting 29, 15,
337. Zerillo, J A; Stuver, S O; Fraile, B; Dodek, A D; Jacobson, J O (2015) Understanding oral chemotherapy prescribing patterns at the end of life at a comprehensive cancer center: Analysis of a Massachusetts payer claims database. Journal of Oncology Practice 11, 5, 372-377
338. Zhong, Y. (2019) Pattern of disease management costs for advanced bladder cancer patients receiving chemotherapy. Journal of Managed Care and Specialty Pharmacy S30
339. Zhu, Y; Tang, K; Zhao, F; Zang, Y; Wang, X; Li, Z; Sun, X; Yu, J (2018) End-of-life chemotherapy is associated with poor survival and aggressive care in patients with small cell lung cancer. Journal of Cancer Research and Clinical Oncology 144, 8, 1591-1599
340. Ziegler, L E; Craigs, C L; West, R M; Carder, P; Hurlow, A; Millares-Martin, P; Hall, G; Bennett, M I (2018) Is palliative care support associated with better quality end-of-life care indicators for patients with advanced cancer? A retrospective cohort study. BMJ Open 8
341. Zimmerman, C T; Shinde, S S; Kasi, P M; Litzow, M R; Huddleston, J M (2016) Patterns of palliative care utilization and end of life care in adult patients with cancer who died as inpatients at Mayo Clinic. Journal of Clinical Oncology. Conference 34

## **References (N=69)**

1. Abdel-Razeq, H.; Shamieh, O.; Abu-Nasser, M.; Nassar, M.; Samhouri, Y.; Abu-Qayas, B.; Asfour, J.; Jarrah, J.; Abdelrahman, Z.; Ameen, Z.; Al-Hawamdeh, A.; Alomari, M.; Al-Tabba, A.; Al-Rimawi, D.; Hui, D. (2019) Intensity of Cancer Care Near the End of Life at a Tertiary Care Cancer Center in Jordan. Journal of Pain and Symptom Management 57, 6, 1106-1113
2. Agarwal, R.; Epstein, A. S.; Saltz, L. B. (2018) Survival outcomes and end of life use of immunotherapy (IO) supplied free of charge for cancer patients. Journal of Clinical Oncology 36, 34,
3. Ang, E; Newton, L V (2018) Thirty-day mortality after systemic anticancer treatment as a real-world, quality-of-care indicator: the Northland experience. Internal Medicine Journal 48, 4, 403-408
4. Assi, T; El Rassy, E; Ibrahim, T; Moussa, T; Tohme, A; El Karak, F; Farhat, F; Faddoul, S; Ghosn, M; Kattan, J (2017) The role of palliative care in the last month of life in elderly cancer patients. Supportive Care in Cancer 25, 2, 599-605
5. Bahler, C; Signorell, A; Blozik, E; Reich, O (2018) Intensity of treatment in swiss cancer patients at the end-of-life. Cancer Management and Research 10, 481-491
6. Balsalobre-Yago, J.; Martínez-Ortiz, M. J.; Alcázar, M. I. L.; Fuentes, P. C.; Checa-Soriano, A.; Viney, A.; Martínez-Penella, M.; Romero, J. L. A.; Ayala, F. (2018) The impact of inclusion in home palliative program and distance to hospital on chemotherapy near end of life. Journal of Clinical Oncology 36, 15,
7. Beck, A. C. (2019) Making treatment decisions at end of life in a comprehensive cancer center. Journal of Clinical Oncology 51
8. Colombet, I.; Bouleuc, C.; Piolot, A.; Vilfaillot, A.; Jaulmes, H.; Voisin-Saltiel, S.; Goldwasser, F.; Vinant, P. (2019) Multicentre analysis of intensity of care at the end-of-life in patients with advanced cancer, combining health administrative data with hospital records: variations in practice call for routine quality evaluation. BMC Palliative Care 18, 1,
9. de Oliveira Valentino, T. C. (2020) Impact of Palliative Care on Quality of End-of-Life Care Among Brazilian Patients With Advanced Cancers. J Pain Symptom Manage 39-48
10. De Schreye, R; Smets, T; Annemans, L; Deliens, L; Gielen, B; De Gendt, C; Cohen, J (2017) Applying quality indicators for administrative databases to evaluate end-of-life care for cancer patients in Belgium. Health Affairs 36, 7, 1234-1243
11. Diaz, L; Jeanpierre, M; Berod, T (2016) Chemotherapy orders near the end of life: A retrospective study in a French Nonteaching Hospital. Pharmacien Hospitalier et Clinicien 51, 1, e1-e5
12. Do, K; Sadeghi, S; Matsuura, P; Lynch, G; Barzi, A (2017) Characteristics of patients (pts) receiving end of life treatment (EOLT) at an NCIDesignated Cancer Center. Journal of Clinical Oncology. Conference 35, 15,
13. Fond, G. (2020) End-of-Life Care Among Patients With Bipolar Disorder and Cancer: A Nationwide Cohort Study. Psychosom Med 722-732
14. Fuchs, Fabiola; Robausch, Martin (2018) [End-of-life therapy for patients dying with cancer: a retrospective database study]. Krebserkrankungen - Therapie am Lebensende: eine retrospektive Datenanalyse. 168, 13, 344-349
15. García Martín, E.; Escudero Vilaplana, V.; Collado Borrell, R.; González-Haba Peña, E.; Marzal Alfaro, B.; Fox, B.; Hoyo Muñoz, Á; Martínez Ortega, P. A.; Amor García, M. Á; Herranz Alonso, A.; Sanjurjo Saéz, M. (2019) Assessment of aggressive care in oncology patients at the end of life in clinical practice. European Journal of Hospital Pharmacy 26, A120-A121
16. Gilbar, Peter J; McPherson, Ian; Aisthorpe, Genevieve G; Kondalsamy-Chennakes, Srinivas (2019) Systemic anticancer therapy in the last 30 days of life: Retrospective audit from an Australian Regional Cancer Centre. Journal of oncology pharmacy practice : official publication of the International Society of Oncology Pharmacy Practitioners 1078155217752077-1078155217752077
17. Hassan, A; Elazzazy, S; Haddad, P (2016) End of life care for haematologic malignancies: A retrospective cohort study from the state of qatar. Supportive Care in Cancer 25, S227-S227
18. Haukland, (2020) Adverse events in deceased hospitalised cancer patients as a measure of quality and safety in end-of-life cancer care. BMC Palliative Care
19. Hiramoto, S.; Tamaki, T.; Nagashima, K.; Hori, T.; Kikuchi, A.; Yoshioka, A.; Inoue, A. (2018) Prognostic factors in patients who received end-of-life chemotherapy for advanced cancer. International Journal of Clinical Oncology 24, 4, 454-459
20. Hirvonen, O. M. (2019) Assessing the utilization of the decision to implement a palliative goal for the treatment of cancer patients during the last year of life at Helsinki University Hospital: a historic cohort study. Acta Oncol 1699-1705
21. Huang, H K; Wang, Y W; Hsieh, J G; Hsieh, C J (2017) Disparity of end-of-life care in cancer patients with and without schizophrenia: A nationwide population-based cohort study. Schizophrenia Research 195, 434-440
22. Jho, H. J. (2020) Changes of End of Life Practices for Cancer Patients and Their Association with Hospice Palliative Care Referral over 2009-2014: A Single Institution Study. Cancer Res Treat 419-425
23. Keating, N. L.; O'Malley, A. J.; Onnela, J. P.; Gray, S. W.; on, B. E. (2019) Influence of Peer Physicians on Intensity of End-of-Life Care for Cancer Decedents. Medical Care 57, 6, 468-474
24. Khaki, A. R. (2020) Comparison of Health Care Utilization at the End of Life Among Patients With Cancer in Alberta, Canada, Versus Washington State. JCO Oncol Pract Op2000217
25. Kim, H.; Keating, N. L.; Perloff, J. N.; Hodgkin, D.; Liu, X.; Bishop, C. E. (2019) Aggressive Care near the End of Life for Cancer Patients in Medicare Accountable Care Organizations. Journal of the American Geriatrics Society 67, 5, 961-968
26. Koroukian, S M; Schiltz, N K; Warner, D F; Given, C W; Schluchter, M; Owusu, C; Berger, N A (2017) Social determinants, multimorbidity, and patterns of end-of-life care in older adults dying from cancer. Journal of Geriatric Oncology 8, 2, 117-124
27. Kraut, J; Gippetti, J; Peterson, D; Agarwala, V; Revol, C; Fessele, K L; Abernethy, A P (2017) Chemotherapy use near end of life (EOL): Measuring real world benchmarks. Journal of Clinical Oncology. Conference 35, 8,
28. Ledoux, M; Tricou, C; Roux, M; Dreano-Hartz, S; Ruer, M; Filbet, M (2018) Cancer Patients Dying in the Intensive Care Units and Access to Palliative Care. Journal of Palliative Medicine 21, 5, 689-693
29. Llabres Valenti, E; Brenes, J; Hernandez, A; Ramchandani Vaswani, A; Beltra, L; Vicente, E; Cejuela, M; Rejas, D (2016) Antineoplastic therapy near the end of life: A retrospective analysis of the clinical practice in oncological adult patients. Annals of Oncology. Conference: 41st European Society for Medical Oncology Congress, ESMO 27
30. Low, D; Merkel, E C; Menon, M; Lyman, G H; Ddungu, H; Namukwaya, E; Leng, M; Casper, C (2017) Chemotherapy Use at the End of Life in Uganda. Journal of global oncology 3, 6, 711-719
31. Mack, J W; Cannavale, K; Sattayapiwat, O; Cheung, B; Chen, L H; Cooper, R M; Chao, C R (2016) Care in the final month of life among adolescent and young adult cancer patients in Kaiser Permanente Southern California. Journal of Palliative Medicine 19, 11, 1136-1141
32. Mallett, V; Linehan, A; Burke, O; Laura, H; Sarah, P; Ryan, K; McCaffrey, J; Kelly, C; Higgins, M (2018) A review of cancer-directed treatments and palliative care provided to solid tumour oncology patients in the 12 weeks preceding death at an Irish University Hospital. Palliative Medicine 32, 10-10
33. McCracken, J A; Dabscheck, A; Coperchini, M; Hornung, I; Jalali, A; Akers, G; Karahalios, A; Gore, F; Lipton, L (2018) Prospective analysis of 30-day mortality following palliative chemotherapy at a tertiary cancer centre. Cancer Reports 1
34. McNaughton, C.; Lynch, S.; Newport, K. B.; Wong, R.; Svetec, S.; Moore, J.; Holliday, R.; Oyer, R. A.; Sivendran, S. (2018) Patterns of anti-cancer therapy use in the last 14 days of life in a community cancer institute. Journal of Clinical Oncology 36, 34,
35. McPherson, J. P.; Patel, S. B.; Igumnova, E.; Pettit, J.; Ose, D.; Haydell, T.; Martineau, J.; Meropol, N. J.; Beck, A. C. (2018) Real-time assessment of resource utilization and subsequent cost analysis in cancer patients (pts) near the end of life (EOL). Journal of Clinical Oncology 36, 30,
36. Morin, L; Beaussant, Y; Aubry, R; Fastbom, J; Johnell, K (2016) Aggressiveness of end-of-life care for hospitalized cancer patients with and without dementia: A nationwide matched cohort study in france. Palliative Medicine 30, NP167-NP167
37. Namireddy, P; Macherla, S; McClain, J T; Muzafiar, M (2017) Racial and social disparities on aggressiveness of end-of-life cancer care in a rural academic center. Journal of Clinical Oncology. Conference 35, 15,
38. Nappa, U; Lindqvist, O; Rasmussen, B H; Axelsson, B (2016) Routine assessment of performance status during palliative chemotherapy when approaching end-of-life. European journal of oncology nursing : the official journal of European Oncology Nursing Society 21, 266-271
39. Newport, K; Holliday, R; McNaughton, C; Gehron, E; Sivendran, M H S (2017) A description of cancer directed therapy within 14 days of death in a community cancer institute. Journal of Clinical Oncology 35, 73-73
40. Nguyen, M; Shum, E; Ng Ying Kin, S; Wann, A; Tamjid, B; Torres, J (2018) Anticancer therapy within the last 30 days of life in a regional cancer centre. Supportive Care in Cancer 26, S385-S385
41. Nguyen, M. (2020) Anticancer therapy within the last 30 days of life: results of an audit and re-audit cycle from an Australian regional cancer centre. BMC Palliat Care 14
42. Paiva, C E; De Oliveira Valentino, T C; Sakamoto, B; Paiva, R (2016) Impact of palliative care (PC) on aggressive end-of-life (EOL) care indicators among advanced cancer patients (ACPs). Journal of Clinical Oncology. Conference 34
43. Paque, K; Elseviers, M; Vander Stichele, R; Pardon, K; Hjermstad, M J; Kaasa, S; Dilles, T; De Laat, M; Van Belle, S; Christiaens, T; Deliens, L (2018) Changes in medication use in a cohort of patients with advanced cancer: The international multicentre prospective European Palliative Care Cancer Symptom study. Palliative Medicine 32, 4, 775-785
44. Parikh, R B; Elffky, A; Pany, M J; Obermeyer, Z (2017) A machine learning approach to predicting short-term mortality risk for patients starting chemotherapy. Journal of Clinical Oncology. Conference 35, 15,
45. Pitson, G.; Matheson, L.; Garrard, B.; Eastman, P.; Rogers, M. (2020) Population-Based Analysis of Radiotherapy and Chemotherapy Treatment in the Last Month of Life within regional Australia. Internal medicine journal
46. Rautakorpi, L K; Seyednasrollah, F; Makela, J M; Hirvonen, O M; Laitinen, T; Elo, L L; Jyrkkio, S M (2017) End-of-life chemotherapy use at a Finnish university hospital: a retrospective cohort study. Acta Oncologica 56, 10, 1272-1276
47. Rochigneux, P; Raoul, J L; Beaussant, Y; Aubry, R; Goldwasser, F; Tournigand, C; Morin, L (2017) Use of chemotherapy near the end of life: What factors matter?. Annals of Oncology 28, 4, 809-817
48. Romano, A M; Gade, K E; Nielsen, G; Havard, R; Harrison, J H; Barclay, J; Stukenborg, G J; Read, P W; Blackhall, L J; Dillon, P M (2017) Early palliative care reduces end-of-life intensive care unit (ICU) use but not ICU course in patients with advanced cancer. Oncologist 22, 3, 318-323
49. Rozman, L M; Campolina, A G; Lopez, R V M; Kobayashi, S T; Chiba, T; De Soarez, P C (2018) Early Palliative Care and Its Impact on End-of-Life Care for Cancer Patients in Brazil. Journal of Palliative Medicine 21, 5, 659-664
50. Sánchez-Cuervo, M. (2020) Chemotherapy Near the End of Life in Onco-Hematological Adult Patients. Am J Hosp Palliat Care 641-647
51. Santos Pérez, M. I. (2019) [Chemotherapy at the end of life: Uncommon clinical practice?]. J Healthc Qual Res 201-207
52. Sato, Y; Miyashita, M; Sato, K; Fujimori, K; Ishikawa, K B; Horiguchi, H; Fushimi, K; Ishioka, C (2018) End-of-life care for cancer patients in Japanese acute care hospitals: A nationwide retrospective administrative database survey. Japanese journal of clinical oncology 48, 10, 877-883
53. Schulkes, K J G; van Walree, I C; van Elden, L J R; van den Bos, F; van Huis-Tanja, L; Lammers, J W J; Ten Bokkel Huinink, D; Hamaker, M E (2018) Chemotherapy and healthcare utilisation near the end of life in patients with cancer. European journal of cancer care 27, 2, e12796-e12796
54. Sedhom, R; Sedhom, D; Barile, D (2017) A comparison of quality end-of-life care in patients with hematologic and solid malignancies: Identifying deficiencies in patient care. Journal of General Internal Medicine 32, S92-S92
55. Sheng, Jin; Zhang, Ya-Xiong; He, Xiao-Bo; Fang, Wen-Feng; Yang, Yun-Peng; Lin, Gui-Nan; Wu, Xuan; Li, Ning; Zhang, Jing; Zhai, Lin-Zhu; Zhao, Yuan-Yuan; Huang, Yan; Zhou, Ning-Ning; Zhao, Hong-Yun; Zhang, Li (2017) Chemotherapy Near the End of Life for Chinese Patients with Solid Malignancies. The oncologist 22, 1, 53-60
56. Sivendran, S.; Lynch, S.; McNaughton, C.; Wong, R.; Svetec, S.; Moore, J. H.; Holliday, R.; Oyer, R. A.; Newport, K. (2019) Anticancer Therapy at the End of Life: Lessons From a Community Cancer Institute. Journal of palliative care 825859719851484
57. Skov Benthien, K; Adsersen, M; Petersen, M A; Soelberg Vadstrup, E; Sjogren, P; Groenvold, M (2018) Is specialized palliative cancer care associated with use of antineoplastic treatment at the end of life? A population-based cohort study. Palliative Medicine 32, 9, 1509-1517
58. Soares, L. G. L. (2020) Quality Indicators of End-of-Life Care Among Privately Insured People With Cancer in Brazil. Am J Hosp Palliat Care 594-599
59. Stuver, S O; McNiff, K; Fraile, B; Odejide, O; Abel, G A; Dodek, A; Jacobson, J O (2016) Novel Data Sharing Between a Comprehensive Cancer Center and a Private Payer to Better Understand Care at the End of Life. Journal of Pain and Symptom Management 52, 2, 161-169
60. Taberner Bonastre, P; Taberner Bonastre, M T; Soler Company, E; Perez-Serrano Lainosa, M D (2016) Chemotherapy near the end of life; assessment of the clinical practise in onco-hematological in adult patients. Farmacia hospitalaria : organo oficial de expresion cientifica de la Sociedad Espanola de Farmacia Hospitalaria 40, 1, 14-24
61. Tancredi, R; Stefani, S; Gervaso, L; Fregoni, V; Poojary, P; Cefali, M; Piacentini, G; Riccardi, A (2017) End-of-life chemotherapy and adherence to ASCO's Top Five List recommendations: A single-center retrospective review. Journal of Clinical Oncology. Conference 35, 15,
62. Tassinari, D; Panzini, I; Fabbri, E; Cherubini, C; Gallegati, D; Rossi, L; Nanni, O; Massa, I; Maltoni, M (2016) Trend in treatment aggressiveness at the end of life in an Italian homogeneous area: Preliminary data. Journal of Clinical Oncology. Conference 34
63. Tsumura, A; Noguchi-Abe, K; Ishida, Y (2018) Quality of end of life care in adolescent and young adults with cancer in Japan. Supportive Care in Cancer 26, S386-S386
64. Urvay, S. (2020) Chemotherapy at the End of Life. J Palliat Care 825859720946505
65. Wang, Shi-Yi; Hall, Jane; Pollack, Craig E; Adelson, Kerin; Bradley, Elizabeth H; Long, Jessica B; Gross, Cary P (2016) Trends in end-of-life cancer care in the Medicare program. Journal of Geriatric Oncology 7, 2, 116-125
66. Webster, J; Scott, J; Andrew Johnson, K (2018) Impact of chemotherapy provided to patients late in life on resource utilization and cost of care. Journal of Clinical Oncology. Conference 36, 15,
67. Wu, Chin-Chia; Hsu, Ta-Wen; Chang, Chun-Ming; Lee, Cheng-Hung; Huang, Chih-Yuan; Lee, Ching-Chih (2016) Palliative Chemotherapy Affects Aggressiveness of End-of-Life Care. The oncologist 21, 6, 771-777
68. Zachariah, F.; Morse, D.; Kinsey, L.; Loscalzo, M.; Koczywas, M.; Dale, W. (2019) The Integrated Care Service: Impact of a Multidisciplinary Supportive Care Service on Hospice Discharge Rates for Medical Oncology Patients in a NCI-Designated Cancer Center (SA511B). Journal of Pain and Symptom Management 57, 2, 448
69. Zimmerman, C T; Shinde, S S; Kasi, P M; Litzow, M R; Huddleston, J M (2016) Patterns of palliative care utilization and end of life care in adult patients with cancer who died as inpatients at Mayo Clinic. Journal of Clinical Oncology. Conference 34
